# Benzopyrazine-Based Small Molecule Inhibitors As Trypanocidal and Leishmanicidal Agents: Green Synthesis, *In Vitro*, and *In Silico* Evaluations

**DOI:** 10.3389/fchem.2021.725892

**Published:** 2021-09-17

**Authors:** Jonathan Rock, Daniel Garcia, Omar Espino, Shaila A. Shetu, Manuel J. Chan-Bacab, Rosa Moo-Puc, Navin B. Patel, Gildardo Rivera, Debasish Bandyopadhyay

**Affiliations:** ^1^ Department of Chemistry, University of Texas Rio Grande Valley, Edinburg, TX, United States; ^2^ Departamento de Microbiología Ambiental y Biotecnología, Universidad Autónoma de Campeche, Campeche, México; ^3^ Unidad Médica de Alta Especialidad, Instituto Mexicano Del Seguro Social, Mérida, México; ^4^ Department of Chemistry, Veer Narmad South Gujarat University, Gujrat, India; ^5^ Laboratorio de Biotecnología Farmacéutica, Centro de Biotecnología Genómica, Instituto Politécnico Nacional, Reynosa, México; ^6^ School of Earth Environment and Marine Sciences (SEEMS), University of Texas Rio Grande Valley, Edinburg, TX, United States

**Keywords:** trypanocidal1, leishmanicidal2, trypanosoma cruzi3, small molecule inhibitors4, quinoxalines5, ecofriendly6, on-water7

## Abstract

World Health Organization (WHO) identified twenty tropical disease categories as neglected tropical diseases (NTDs)^1^. Chagas’ disease (also known as American trypanosomiasis) and leishmaniasis are two major classes of NTDs. The total number of mortality, morbidity, and disability attributed each year due to these two categories of diseases in magnitudes is much higher than the so-called elite diseases like cancer, diabetes, AIDS, cardiovascular and neurodegenerative diseases. Impoverished communities around the world are the major victim of NTDs. The development of new and novel drugs in the battle against Chagas’ disease and leishmaniasis is highly anticipated. An easy and straightforward on-water green access to synthesize benzopyrazines is reported. This ultrasound-assisted procedure does not require any catalyst/support/additive/hazardous solvents and maintains a high atom economy. A series of eleven benzopyrazines has been synthesized, and most of the synthesized compounds possess the drug-likeness following Lipinski’s “Rule of 5”. Benzopyrazines **3** and **4** demonstrated moderate leishmanicidal activity against *L. mexicana* (M378) strain. The selective lead compound **1** showed good leishmanicidal, and trypanocidal activities (*in vitro*) against both *L. mexicana* (M378) and *T. cruzi* (NINOA) strains compared to the standard controls. The *in vitro* trypanocidal and leishmanicidal activities of the lead compound **1** have been validated by molecular docking studies against four biomolecular drug targets *viz. T. cruzi* histidyl-tRNA synthetase, *T. cruzi trans*-sialidase, leishmanial rRNA A-site, and leishmania major *N*-myristoyl transferase.

## Introduction

**FIGURE 1:**
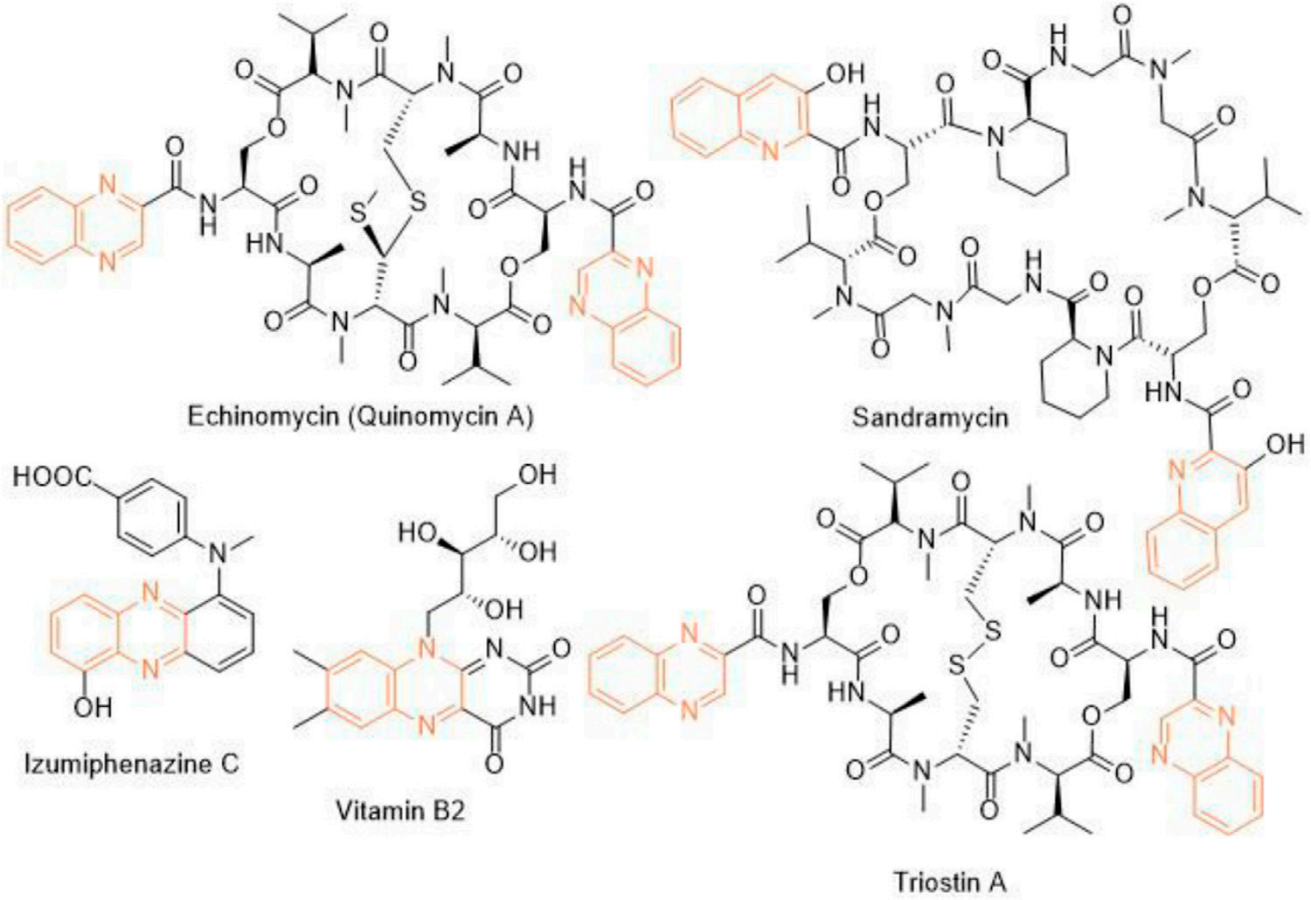
Representative examples of naturally occurring bioactive benzopyrazines.

Heterocycles play a significant role in drug discovery research. A considerable number of the small molecule inhibitors are heterocyclic compounds. Among various classes of heterocycles, for example, aza-, oxo-, phospho-, thioheterocycles; aza- or nitrogen heterocycles are found in many pharmacologically relevant compounds natural and synthetic. Benzopyrazine (also known as quinoxaline) is a fused bicyclic scaffold in which benzene is fused with pyrazine (Bandyopadhyay and Banik, 2021). A few naturally occurring antibiotics and nutrients like vitamin B2 contain benzopyrazine moiety as a core in their structures (Figure 1). This scaffold is present as the key structural motif in many biologically active compounds, which includes anticancer (Lee et al., 2012; Bandyopadhyay et al., 2013; Qi et al., 2018), antibacterial (El-Attar et al., 2018), antitubercular (Achutha et al., 2013), anti-ebola (Loughran et al., 2016), antifungal (Carta et al., 2001) among many others. Consequently, several procedures of synthesizing benzopyrazines have been reported in the literature. A few synthetic procedures, reported in the immediate past, include the synthesis of benzopyrazines under the catalytic influence of Co_3_O_4_ nanocages based nickel catalyst (Sharma et al., 2021), iron-catalyzed transfer hydrogenative condensation (Putta et al., 2021), sodium hydroxide-mediated hydrogen-transfer (Wang et al., 2021), as well as the uses of tungstophosphoric acid-support (Kumaresan et al., 2020), and Co-based nanocatalyst (Panja et al., 2020).

**SCHEME 1:**
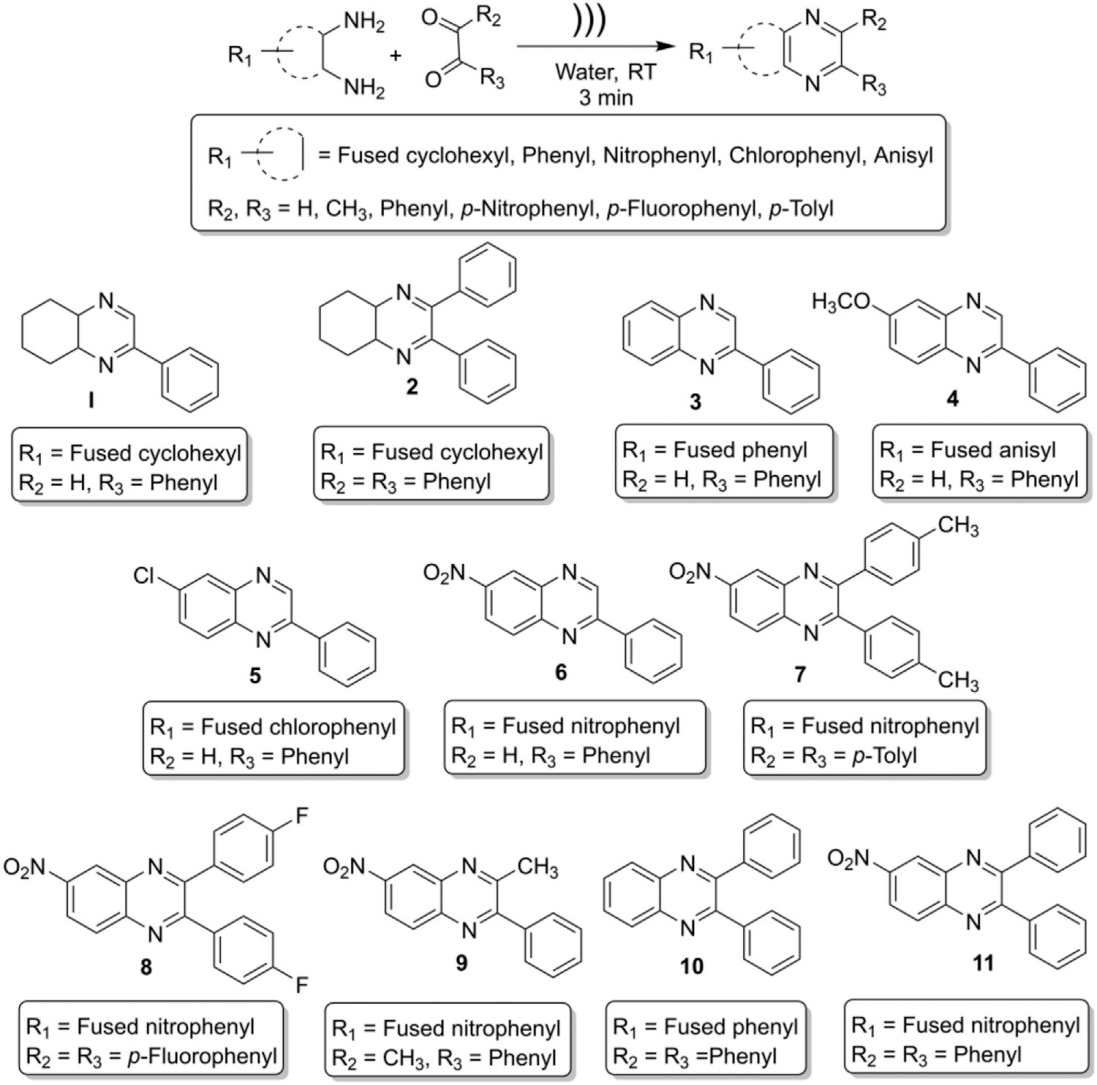
Ultrasound-assisted on-water green synthesis of diverse benzopyrazines.

On the other hand, the World Health Organization (WHO) identified twenty tropical disease categories as neglected tropical diseases (NTDs). In general, the population below the poverty line are the primary sufferers of NTDs. Every year millions of people from 149 countries worldwide are being infected by NTDs that cause the waste of billions of dollars and a loss of thousands of lives. Based on the mortality and morbidity rates, Chagas’ disease (named after the Latin American physician Carlos Chagas, also known as American trypanosomiasis) and leishmaniasis are two major categories of NTDs that demand immediate attention from the global community (Maheshwari and Bandyopadhyay, 2021). We report herein an ultrasound-assisted on-water green synthesis of diversely substituted benzopyrazine derivatives (Scheme 1) and subsequent *in vitro* trypanocidal and leishmanicidal evaluation of these compounds. Further, we hypothesize that the biological (trypanocidal and leishmanicidal) activity is due to the interaction and subsequent inhibition of the protozoal proteins responsible for these diseases. In addition, we validated our hypothesis through *in silico* docking study.

## Materials and Methods

### General

We determined the melting points of the final products by a digital melting point apparatus (DigiMelt MPA 160 by SRS). Elemental (CHN) analyses were carried out by a PerkinElmer 2,400 Series II elemental analyzer, their results were found to be in good agreement (±0.5%) with the calculated values. Sonication was performed with the UP200St (200W, 26kHz) ultra-sonicator (Hielscher Ultrasonics GmbH, Germany). FT-IR spectra were recorded on a Bruker Alpha modular Platinum-ATR FT-IR spectrometer with OPUS software, using the samples directly (neat) without making pallets. ^1^H NMR (600 MHz) and ^13^C NMR (150 MHz) spectra were obtained at room temperature with Bruker superconducting Ultrashield Plus 600 MHz NMR spectrometer with central field 14.09 T, coil inductance 89.1 Hz, and magnetic energy 1,127.2 kJ using CDCl_3_ or d_6_-DMSO as solvent. Chemicals were purchased from Sigma-Aldrich, Inc. (St. Louis, MO) and VWR International (Missouri, TX). Solvents were purchased from Fisher Scientific International Inc. (Pittsburgh, PA) throughout the investigation.

### General Procedure for the Synthesis of Benzopyrazines

In a general procedure of this on-water reaction, *ortho*-diamine and dicarbonyl compounds were mixed (1:1 M ratio) in a hard glass test tube, and 1 ml tap water was added to the reaction mixture. In a model reaction, 1 ml water was taken in a hard glass test tube, and 1 mmol of *o*-phenylene diamine (108 mg) and 1 mmol of phenylglyoxal hydrate (134 mg) were added into the test tube. The ultrasonic probe was inserted into the test tube (reaction mixture), and the mixture was sonicated. The reaction was monitored by thin-layer chromatography (TLC) every after 1 minute. The most intense TLC spot was seen after 3 minutes. Further sonication did not increase the intensity of the product spot; rather, it reduced the product and generated gummy material. Initially, the reaction was carried out in deionized water, but no significant change in yield was observed compared to tap water. At the end of the reaction, the product looked like a chunk, and it was not soluble in water. Consequently, the product was extracted with 9 ml (3 × 3 mL) of ethyl acetate. The organic layer was dried over sodium sulfate and concentrated by reduced pressure distillation by a rotary evaporator. The crude mass was passed quickly through a purification column with ethyl acetate to get highly pure (>98%) and crystalline compounds. The spectral data of the compounds are given below:

*2-phenyl-4a,5,6,7,8,8a-hexahydroquinoxaline* (***1,*** Scheme 1)*.* Brownish-yellow amorphous solid (206 mg, 97%); m. p. 135–136°C; IR (KBr) 3,415, 1,660, 1,536, 1,448, 1,294, 764 cm^−1^; ^1^H NMR (600 MHz, CDCl_3_) d 1.46–2.18 (m, 8H), 3.66 (br s, 1H), 3.77 (d, *J =* 3.60 Hz, 1H), 7.42 (s, 1H), 7.48–7.50 (m, 2H), 7.86 (dd, *J =* 7.56, 1.38 Hz, 2H), 8.39 (d, *J =* 2.10 Hz, ^1^H); ^13^C NMR (150 MHz, CDCl_3_) 22.35, 22.66, 27.75, 28.07, 54.11, 54.21, 126.59, 128.77, 130.55, 136.12, 151.62, 155.44. Anal. Calcd for C_14_H_16_N_2_: C, 79.21; H, 7.60; N, 13.20. Found: C, 79.09; H, 7.52; N, 13.11.

*2,3-diphenyl-4a,5,6,7,8,8a-hexahydroquinoxaline* (***2,*** Scheme 1)*.* Greenish-yellow crystalline solid (276 mg, 96%); m. p. 94–95°C; IR (KBr) 3,312, 3,061, 2,930, 2,851, 1,665, 1,560, 1,489, 1,446, 1,240, 1,184, 1,077, 1,012, 758, 698 cm−1; ^1^H NMR (600 MHz, CDCl_3_) d 1.48–1.85 (m, 8H), 3.84 (br s, 2H), 7.24–7.39 (m, 10H); ^13^C NMR (150 MHz, CDCl3) 22.51, 27.42, 55.01, 127.92, 128.01, 129.26, 138.29, 159.49. Anal. Calcd for C_20_H_20_N_2_: C, 83.30; H, 6.99; N, 9.71. Found: C, 83.18; H, 6.87; N, 9.58.

*2-Phenylquinoxaline* (***3,*** Scheme 1)*.* Light brown crystalline solid (194 mg, 94%); m. p. 77–79°C; IR (ν in cm^−1^): 1727, 1,538, 1,482, 1,303, 1,124, 1,027, 951, 760, 682, 550; ^1^H NMR (600 MHz, CDCl_3_) δ 7.52–7.59 (m, 3H), 7.74–7.80 (m, 2H), 8.14 (d, *J* = 8.16 Hz, 1H), 8.17 (d, *J* = 8.22 Hz, 1H), 8.21 (d, *J* = 7.62 Hz, 2H), 9.34 (s, 1H); ^13^C NMR (150 MHz, CDCl_3_) δ 127.57, 129.14, 129.16, 129.54, 129.65, 130.20, 130.28, 136.80, 141.60, 142.32, 143.37, 151.85. Anal. Calcd for C_14_H_10_N_2_: C, 81.53; H, 4.89; N, 13.58. Found: C, 81.18; H, 4.87; N, 14.06.

*6-Methoxy-2-phenylquinoxaline* (***4,*** Scheme 1)**:** White crystalline solid (217 mg, 92%); m. p. 75–77°C; IR (ν in cm^−1^): 1,616, 1,541, 1,508, 1,489, 1,456, 1,374, 1,314, 1,212, 1,117, 1,019, 827, 754, 687; ^1^H NMR (600 MHz, CDCl_3_) δ 3.90 (s, 3H), 7.30 (dd, *J* = 8.95, 2.76 Hz, 1H), 7.35 (d, *J* = 2.70 Hz, 1H), 7.43–7.49 (m, 3H), 7.90 (d, *J* = 9.06 Hz, 1H), 8.07 (s, 1H), 8.08 (m, 1H), 9.08 (s, 1H); ^13^C NMR (150 MHz, CDCl_3_) δ 55.84, 106.91, 122.89, 127.52, 129.10, 129.12, 130.05, 137.02, 137.80, 140.76, 143.98, 151.95, 161.08. Anal. Calcd for C_15_H_12_N_2_O: C, 76.25; H, 5.12; N, 11.86. Found: C, 77.01; H, 4.91; N, 12.13.

*6-Chloro-2-phenylquinoxaline* (***5,*** Scheme 1)*:* Light brown crystalline solid (215 mg, 95%); m. p. 146–147°C; IR (ν in cm^−1^): 1,699, 1,540, 1,481, 1,449, 1,315, 1,223, 1,132, 1,073, 958, 829, 756, 711, 687; ^1^H NMR (600 MHz, CDCl_3_) δ 7.47–7.51 (m, 3H), 7.61 (dd, *J* = 8.85, 2.22 Hz, 1H), 7.98 (d, *J* = 8.82 Hz, 1H), 8.01–8.04 (m, 1H), 8.08 (d, *J* = 2.16 Hz, 1H), 8.10–8.12 (m, 1H), 9.24 (s, 1H); ^13^C NMR (150 MHz, CDCl_3_) δ 127.54, 127.63, 129.24, 130.37, 130.46, 130.54, 130.57, 136.34, 140.12, 142.68, 143.44, 152.59. Anal. Calcd for C_14_H_9_ClN_2_: C, 69.86; H, 3.77; N, 11.64. Found: C, 70.23; H, 4.06; N, 10.88.

*6-Nitro-2-phenylquinoxaline* (***6,*** Scheme 1)*:* Pale yellow crystalline solid (246 mg, 98%); m. p. 210–211°C; IR (ν in cm^−1^): 1,616, 1,556, 1,522, 1,348, 1,316, 1,077, 851, 832, 791, 764, 691; ^1^H NMR (600 MHz, CDCl_3_) δ 7.52–7.55 (m, 3H), 8.16–8.21 (m, 3H), 8.47 (dd, *J* = 9.12, 2.52 Hz, 1H), 8.95 (d, *J* = 2.46 Hz, 1H), 9.42 (s, 1H); ^13^C NMR (150 MHz, CDCl_3_) δ 123.79, 125.67, 127.95, 129.43, 131.19, 131.41, 135.64, 140.36, 144.93, 145.50, 147.47, 154.33. Anal. Calcd for C_14_H_9_ClN_2_: C, 69.86; H, 3.77; N, 11.64. Found: C, 70.23; H, 4.06; N, 10.88.

*6-Nitro-2,3-di-p-tolylquinoxaline* (***7,*** Scheme 1)*:* Yellow crystalline solid (352 mg, 99%); m. p. 162–163°C; IR (ν in cm^−1^): 1,521, 1,339, 1,182, 1,050, 978, 818, 723, 601, 545, 530; ^1^H NMR (600 MHz, CDCl_3_) δ 2.41 (s, 6H), 7.21 (d, *J* = 7.86 Hz, 4H), 7.49 (t, *J* = 7.80 Hz, 4H), 8.27 (d, *J* = 9.12 Hz, 1H), 8.50–8.52 (m, 1H), 9.05 (d, *J* = 2.46 Hz, 1H); ^13^C NMR (150 MHz, CDCl_3_) δ 21.42, 21.43, 123.02, 125.54, 129.19, 129.74, 129.84, 130.61, 135.38, 135.43, 139.83, 139.87, 140.03, 143.56, 147.70, 155.70, 156.32. Anal. Calcd for C_22_H_17_N_3_O_2_: C, 74.35; H, 4.82; N, 11.82. Found: C, 73.98; H, 4.53; N, 12.01.

*2,3-Bis(4-fluorophenyl)-6-nitroquinoxaline* (***8,*** Scheme 1)*:* Yellowish solid (337 mg, 93%); m. p. 157–159°C; IR (ν in cm^−1^): 1,599, 1,528, 1,512, 1,397, 1,341, 1,230, 1,163, 1,050, 978, 846, 726, 564; ^1^H NMR (600 MHz, CDCl_3_) δ 7.11 (t, *J* = 8.46 Hz, 4H), 7.56–7.60 (m, 4H), 8.29 (d, *J* = 9.12 Hz, 1H), 8.55 (dd, *J* = 9.12, 2.46 Hz, 1H), 9.06 (d, *J* = 2.46 Hz, 1H); ^13^C NMR (150 MHz, CDCl_3_) δ 115.74, 115.89, 123.51, 125.54, 130.72, 131.81, 131.91, 131.97, 132.02, 133.95, 133.98, 134.02, 134.04, 139.92, 143.48, 148.01, 154.37, 154.97, 162.86, 164.61. Anal. Calcd for C_20_H_11_F_2_N_3_O_2_: C, 66.12; H, 3.05; N, 11.57. Found: C, 66.58; H, 3.07; N, 11.33.

*3-Methyl-6-nitro-2-phenylquinoxaline* (***9,*** Scheme 1)*:* Light yellow crystalline solid (257 mg, 97%); m. p. 131–133°C; IR (ν in cm-1): 1,519, 1,398, 1,328, 1,071, 1,003, 897, 818, 764, 741, 693, 588; ^1^H NMR (600 MHz, CDCl_3_) δ 2.78 (s, 3H), 7.49 (t, *J* = 1.56 Hz, 3H), 7.61 (d, *J* = 5.58 Hz, 2H), 8.10–8.17 (m, 1H), 8.40–8.44 (m, 1H), 8.94 (s, 1H); ^13^C NMR δ (150 MHz, CDCl_3_) 23.85, 12,211, 124.67, 127.73, 127.91, 127.93, 128.72, 128.99, 136.88, 138.82, 142.64, 155.32, 156.15. Anal. Calcd for C_15_H_11_N_3_O_2_: C, 67.92; H, 4.18; N, 15.84. Found: C, 68.05; H, 4.10; N, 15.79.

*2,3-Diphenylquinoxaline* (***10,*** Scheme 1)*:* Colorless crystals (271 mg, 96%); m. p. 118–121°C; IR (ν in cm^−1^): 1,665, 1,560, 1,489, 1,240, 1,184, 1,077, 1,012, 877, 807, 758, 698; ^1^H NMR (600 MHz, CDCl_3_) δ 7.36–7.40 (m, 6H), 7.55 (m, 4H), 7.79 (dd, *J* = 4.86, 3.00 Hz, 2H), 8.21 (dd, *J* = 4.86, 2.88 Hz, 2H); ^13^C NMR (150 MHz, CDCl_3_) δ 128.30, 128.83, 129.24, 129.89, 129.98, 139.13, 141.27, 153.49. Anal. Calcd for C_20_H_14_N_2_: C, 85.08; H, 5.00; N, 9.92. Found: C, 84.97; H, 5.11; N, 10.02.

*6-Nitro-2,3-diphenylquinoxaline* (***11,*** Scheme 1)*:* Deep yellow solid (321 mg, 98%); m. p. 182–184°C; IR (ν in cm^−1^): 1,520, 1,341, 1,188, 1,063, 1,024, 979, 698; ^1^H NMR (600 MHz, CDCl_3_) δ 7.38–7.46 (m, 6H), 7.57–7.60 (m, 4H), 8.31 (d, *J* = 9.18 Hz, 1H), 8.54 (dd, *J* = 2.46, 9.12 Hz, 1H), 9.09 (d, *J* = 2.52 Hz, 1H); ^13^C NMR (150 MHz, CDCl_3_) δ 123.30, 125.64, 128.47, 129.65, 129.84, 129.92, 130.77, 138.05, 138.12, 139.99, 143.59, 147.90, 155.70, 156.32. Anal. Calcd for C_20_H_13_N_3_O_2_: C, 73.38; H, 4.00; N, 12.84. Found: C, 73.69; H, 4.06; N, 12.71.

### Trypanocidal and Leishmanicidal (*in vitro*) Evaluations

We used the promastigotes of *L*. *mexicana* (MHOM/MX/ISETGS) clinical strain for the leishmanicidal growth inhibition assay. The clinical strain was initially isolated from a patient suffering from diffuse cutaneous leishmaniasis. We carried out the trypanocidal assay with the epimastigotes of *T*. *cruzi* (MHOM/MX/1994/NINOA). The clinical strain was originally isolated from a patient with the disease in the acute phase for this assay. Schneider’s *Drosophila* medium, supplemented with 10% fetal bovine serum, penicillin (100 IU/ml), and streptomycin (100 μg/ml), was used to culture the parasites at 26°C. The antiprotozoal assays were carried out in 96-well plates using dimethyl sulfoxide (DMSO) as the carrier. The standard controls and the testing compounds were solubilized in DMSO and diluted as required. All the antiprotozoal assays were performed in duplicate. Aliquots of 100 µL of compound solution and 100 µL of culture medium containing 10,000 *Leishmania* promastigotes or 20,000 *T*. *cruzi* epimastigotes were combined to obtain concentrations of 50, 25, 12.5, 6.25, 3.125 μg/ml, and so on. Two first-line commercial drugs nifurtimox (antichagasic drug) and miltefosine (leishmanicidal drug), were used as positive controls. Only the parasite-containing culture was used as the negative control. The plates were incubated for 72 h at a temperature of 26°C, and the antiprotozoal activity of the compounds was determined by direct count of parasites in a Neubauer chamber (Chan-Bacab et al., 2009). The IC_50_ values (the concentration required to inhibit 50% of parasite growth were calculated (in µg/mL) by probit analysis (Hernández-Núñez et al., 2009).

### *In Silico* Molecular Docking Studies

We conducted the *in silico* molecular docking study following our previously published procedure (Laskar et al., 2019). In brief, the ligands were prepared with their corresponding assigned atoms types and charges using ChemOffice 2015 as MOL files and converted to PDB and PDBQT sequentially following a few steps utilizing open-source and well-known graphic user interface software. We imported the control drugs (positive controls) from ChemSpider as MOL format; if not, control ligands were manually prepared from scratch. Crystal structures of the four proteins 4YPF, 1S0J, 4K32, 6QDA (biological targets in this study) were imported from RCSB (Rose et al., 2011) (http://www.rcsb.org), a member of Worldwide Protein Data Bank (wwPDB). The MOL formats of the ligands were changed to PDB format by Avogadro software (Hanwell et al., 2012). The ligands were saved in. pdb format and AutoDock4. AutoDockTools 1.5.6, part of AutoDock4 (AD4), were used to alter the ligands and receptors (proteins) from PDB to PDBQT formats. PDBQT format provides the molecular structure coordinate files, including atomic partial charges, atom types, torsional flexibility information, etc. The ligand files (in.pdb form) were loaded onto the AutoDockTools dashboard to detect torsion root, rotatable bonds and add gasteiger charges for atomic charges, if necessary. The receptor-binding sites were localized using the Adaptive Poisson-Boltzmann Solver (APBS) plugin for surface electrostatic calculation and Computed Atlas Surface Topology of proteins (CASTp) for a pocket that void detection on the protein surface (Liang et al., 1998). Binding site visualization was made feasible by Schrödinger Maestro, AutoDock4, PyMOL, and other software. (Morris et al., 2009; Seeliger and de Groot, 2010; Schrodinger Release 2016-4, 2016; PyMOL Molecular Graphics System, 2015; Pettersen et al., 2004). These software packages offer a complete molecular viewer and graphic user interface, which are essential for structure-based drug design and discovery. PDBQT structure formats are compatible with AutoDock Vina (Trott and Olson, 2010), a complementary AD4 molecular docking program, and are required to run the docking simulations. We used the Schrödinger Maestro to visualizing and preparing the PDB receptor and ligand conformation files for docking. We uploaded the receptor files to the Maestro workspace, and the binding site surface area was calculated using the Task Tree search bar and selecting Binding Surface Area Analysis. The resulting free energy binding affinities (in kcal/mol) were recorded as docking scores. These scores were considered to evaluate the strength of non-covalent interaction between different receptor-ligand conformations. A conformer with the lowest energy and highest cluster counts was regarded as the best ligand conformation that fits the binding site. The structural analysis was effectuated in Schrödinger Maestro was set to 1) visualize the formation and quantify distances of H-bonds, π-π stacking interactions, and close contacts of each ligand to the receptor of interest; 2) obtain images of ligand-receptor residue interactions within the vicinity of the binding site, and 3) observe the receptor surface homology through molecular dynamics simulation (Haile, 1992). The *in silico* docking study evaluated miltefosine and nifurtimox, leishmanicidal, and trypanocidal control drugs, respectively.

## Results

### Green Synthesis of Diverse Benzopyrazines

Ultrasound-assisted diversely substituted benzopyrazines were synthesized, inserting a powerful sonicator probe into the reaction mixture. For the diamine and dicarbonyl compounds, various substituents based on electron-withdrawing or electron-donating ability were tested to generalize the reaction. The nitro, chloro, on the diamine represent strong and weak electron-withdrawing groups (EWG), whereas the fused cyclohexyl and methoxy groups represent mild and strong electron-donating groups (EDG). The ultimate goal was to evaluate whether the presence of these functional groups influences the nucleophilicity of the diamines. Similarly, the dicarbonyls’ electrophilicity was varied by introducing *p*-nitro, *p*-fluoro (EWG) or methyl (EDG) in the dicarbonyl system. The overall yield of the products indicates that the presence of EWG or EDG does not influence the reaction significantly, which in turn supports the universality of this synthetic method. Based on the available literature, this is the first example of synthesizing benzopyrazines under this condition. Our method produced excellent yields of the corresponding products (overall yield 92% or more) in a very short period. The yield of the products and the atom economy of the reactions are shown in Table 1. The range of atom economy in the series varies from 76.92 to 88.89%, which undoubtedly supports the greenness of the synthetic method.

**TABLE 1:** The yield, and atom economy in the synthesis of benzopyrazines (**1**–**11**).

Compound	Yield (%)^a^	Atom economy
**1**	97	85.48
**2**	96	88.89
**3**	94	76.92
**4**	92	80.00
**5**	95	76.92
**6**	98	78.57
**7**	99	86.36
**8**	93	84.21
**9**	97	80.00
**10**	96	83.33
**11**	98	84.21

a Isolated yield.

### Trypanocidal and Leishmanicidal Evaluations (*in vitro*) of the Benzopyrazines (1–11)

We used the promastigotes of *L*. *mexicana* (MHOM/MX/ISETGS) clinical strain for the leishmanicidal growth inhibition assay. The clinical strain was initially isolated from a patient suffering from diffuse cutaneous leishmaniasis. We carried out the trypanocidal assay with the epimastigotes of *T*. *cruzi* (MHOM/MX/1994/NINOA). The clinical strain was originally isolated from a patient with the disease in the acute phase for this assay. Schneider’s *Drosophila* medium, supplemented with 10% fetal bovine serum, penicillin (100 IU/ml), and streptomycin (100 μg/ml), was used to culture the parasites at 26 °C. Two commercial drugs, miltefosine, and nifurtimox were used as standard controls. Compound **1** demonstrated good *in vitro* activity against both the strains with comparable IC_50_ values with both the controls: miltefosine and nifurtimox. Compounds **3** and **4** showed moderate IC_50_ values against the *L. mexicana* (M378) strain (Table 2). The standard biosecurity and institutional procedure were followed during the experiment.

**TABLE 2:** IC_50_ (µM ± SD) of the benzopyrazine (**1**–**11**) against epimastigotes from *T. cruzi* and promastigote from *L. mexicana*.

Compound	*L. mexicana (M378)*	*T. cruzi (NINOA)*
**1**	12.46 ± 0.62	37.85 ± 0.52
**2**	>100	>100
**3**	50.86 ± 1.02	>100
**4**	39.83 ± 0.29	>100
**5**	>100	>100
**6**	>100	>100
**7**	>100	>100
**8**	>100	>100
**9**	>100	>100
**10**	>100	>100
**11**	>100	>100
Miltefosine	—	19.56 ± 0.61
Nifurtimox	9.32 ± 0.31	—

### *In Silico* Molecular Docking of the Compound 1

**FIGURE 2:**
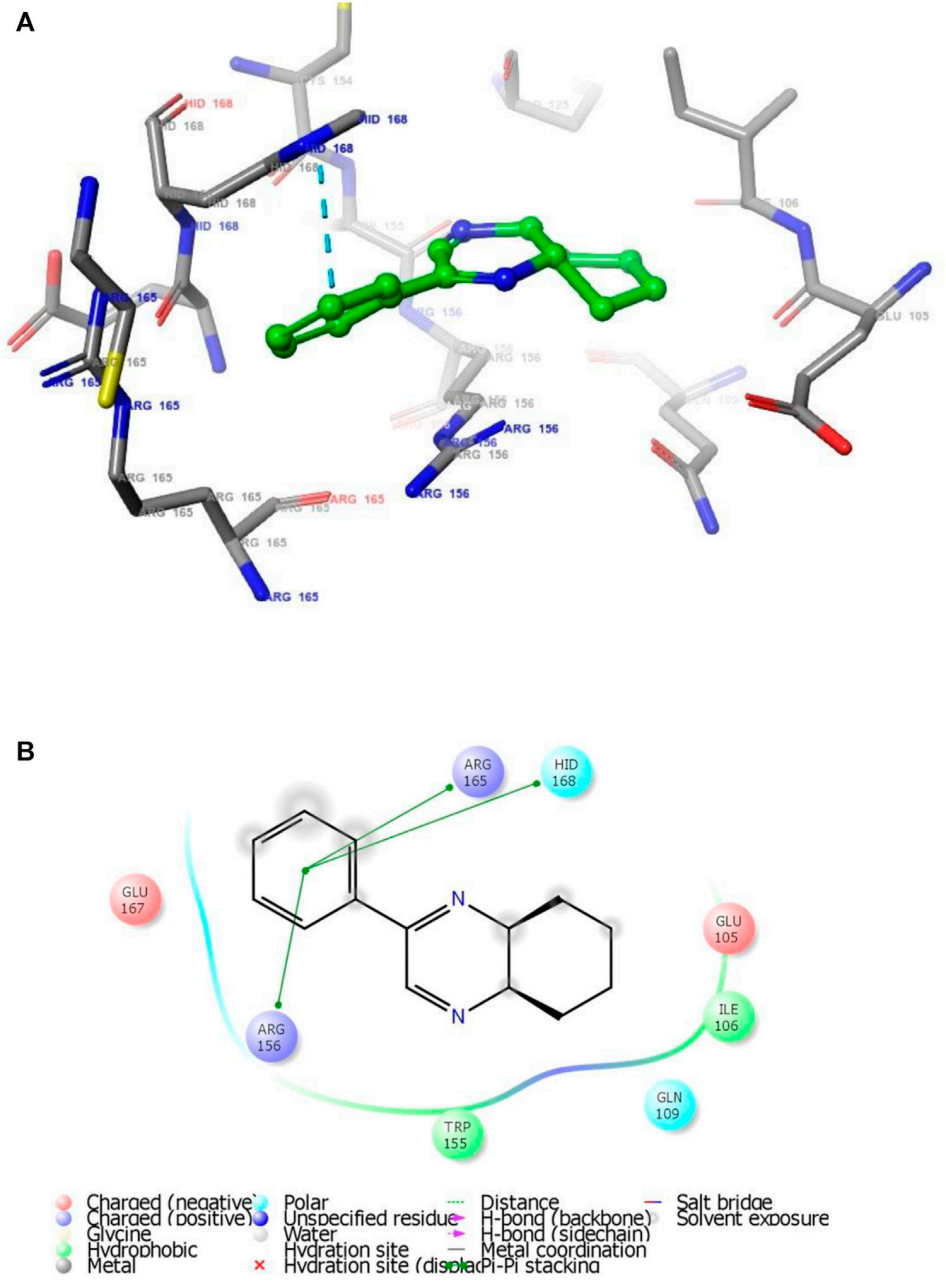
**(A)** The binding mode of the interactions between **1** with *T. cruzi* Histidyl-tRNA synthetase (PDB ID: 4YPF). **(B)** Results of the validation of **1** inside the *T. cruzi* histidyl-tRNA synthetase active sites.

**FIGURE 3:**
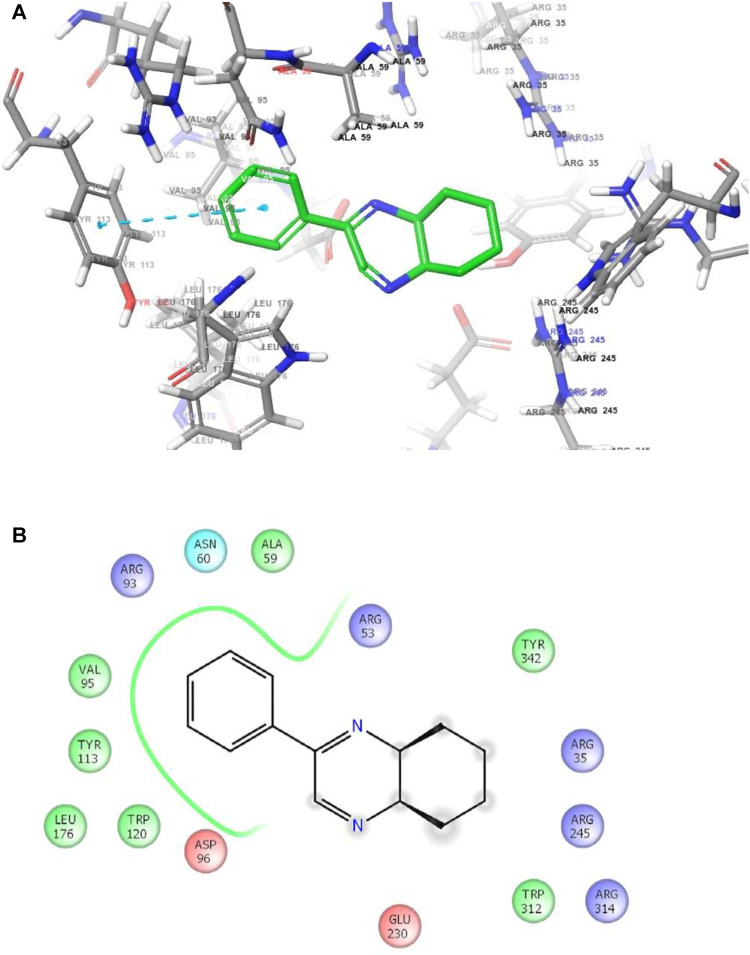
**(A)** The binding mode of the interactions between **1** with *T. cruzi trans*-sialidase (PDB ID: 1S0J). **(B)** Results of the validation of **1** inside the *T. cruzi trans*-sialidase active sites.

**FIGURE 4:**
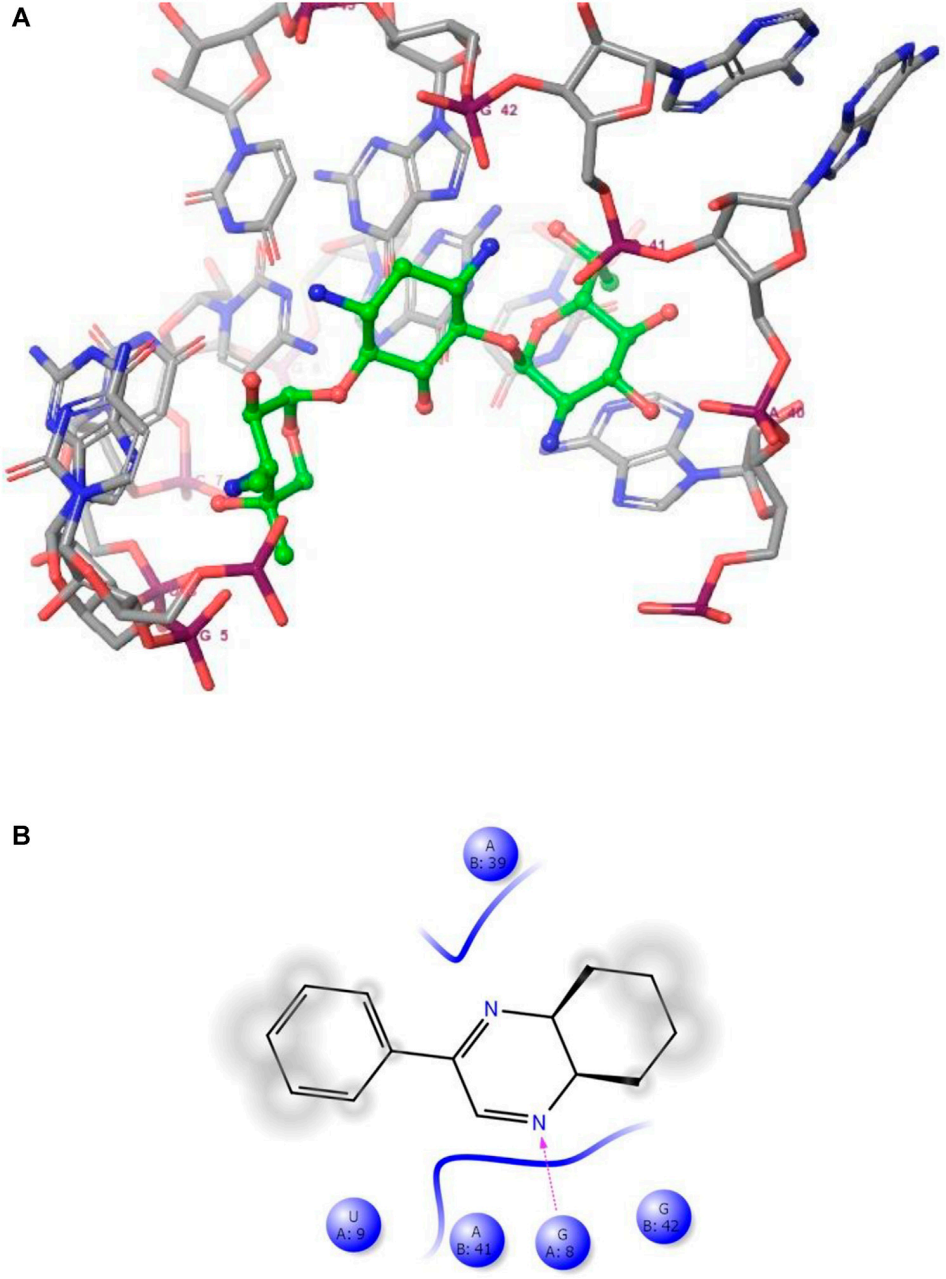
**(A)** The binding mode of the interactions between **1** with Leishmanial rRNA A-site (PDB ID: 4K32). **(B)** Results of the validation of **1** inside the Leishmanial rRNA A-site active sites.

**FIGURE 5:**
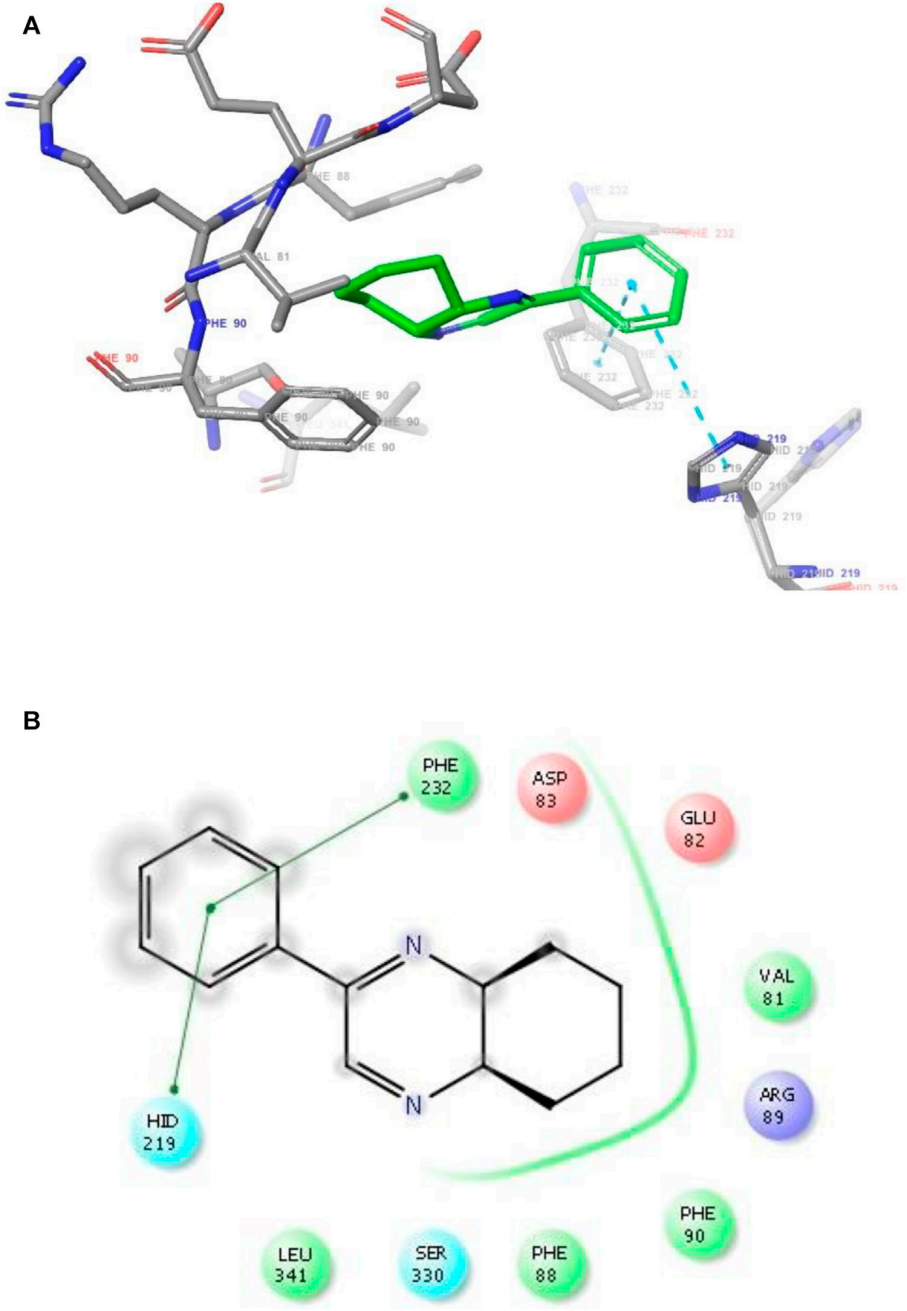
**(A)** The binding mode of the interactions between **1** with Leishmania major *N*-myristoyltransferase (PDB ID: 6QDA). **(B)** Results of the validation of **1** inside the Leishmania major *N*-myristoyltransferase active sites.

*T. cruzi* Histidyl-tRNA synthetase (PDB ID: 4YPF; Koh et al., 2015) and *T. cruzi trans*-sialidase (PDB ID: 1S0J; Amaya et al., 2004) are considered as two major biological drug targets for American trypanosomiasis whereas Leishmanial rRNA A-site (PDB ID: 4K32; Shalev et al., 2013) and Leishmania major *N*-myristoyltransferase (PDB ID: 6QDA; Bell et al., 2020) are considered as the major biomolecular drug targets for leishmaniasis (Kelly et al., 2020). As shown in Table 2, compound 1 showed comparable *in vitro* trypanocidal and leishmanicidal activities with two standard commercial drugs; therefore, we hypothesized the inhibition of the proteins as mentioned earlier with compound 1. To validate our hypothesis, we carried out extensive molecular docking studies of compound 1 against the four proteins. The docking scores are presented in Table 3. Docking interactions between compound 1 with the four proteins are shown in Figures 2–5.

**TABLE 3:** Molecular docking scores of the compound **1** and the standard controls with the biomolecular targets.

Compound	Structure	Docking score^a^ (PDB ID: 4YPF)	Docking score^a^ (PDB ID: 1S0J)	Docking score^a^ (PDB ID: 4K32)	Docking score^a^ (PDB ID: 6QDA)
**1**	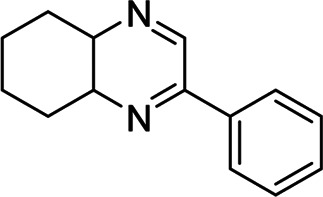	−6.7	−7.3	−6.3	−7.4
Miltefosine	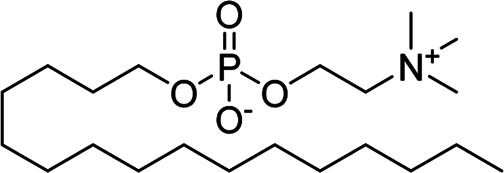	−4.7	−5.4	−4.9	−6.0
Nifurtimox	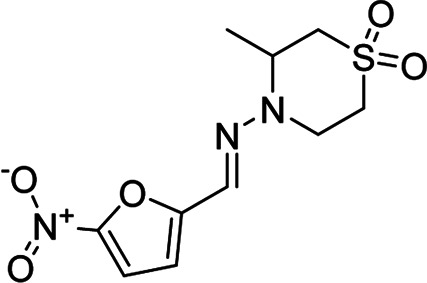	−7.0	−7.8	−6.6	−7.5

a Binding affinity [kcal/mol].

4YPF: Crystal structure of *T. cruzi* Histidyl-tRNA synthetase in complex with quinolin-3-amine.

1S0J: *Trypanosoma cruzi trans*-sialidase in complex with MuNANA (Michaelis complex).

4K32: Crystal structure of geneticin bound to the leishmanial rRNA A-site.

6QDA: Leishmania major *N*-myristoyltransferase in complex with quinazoline inhibitor IMP-0000811.

### *In Silico* Drug-likeness Determination

Drug-likeness indicates the possibility of a molecule becoming a drug. Accordingly, a drug molecule should have a balance of various physicochemical properties like molecular weight, hydrogen bond donor, hydrogen bond acceptor, total polar surface area, rotatable bond, hydrophilicity, lipophilicity, hydrophobicity, lipophobicity, bioavailability, half-life, etc. The druggability assessment of all the eleven benzopyrazines was performed in compliance with Lipinski’s rule of five (RO5) (Lipiniki et al., 1997; Lipiniki et al., 2001; Lipinski, 2004). The druggability parameters of the eleven benzopyrazines are shown in Table 4. Compound 1 shows the minimum miLogP value (2.96) out of all the eleven analogs without having any significant violations of druggability.

**TABLE 4:** Interactions between compound **1** with the four biomolecular targets (PDB IDs: 4YPF, 1S0J, 4K32 and 6QDA).

Receptor	Binding site residue(s)	Type of interactions	Hydrophobic residues	Docking score^a^
4YPF	HID168	π-π stacking	GLU105, ILE106, GLN109, PRO125, TRP155, ARG165, GLU167, CYS365	−6.7
1S0J	N/A	Hydrophobic interaction	ARG35, ARG53, ASN60, ARG93, ASP96, TYR113, TRP120, LEU176, GLU230, TRP312, ARG314, TYR342	−7.3
4K32	Guanine 8	H-Bond	U9, A39, A40, A41, G43	−6.3
6QDA	HID 219, PHE232	π-π stacking	VAL81, GLU82, ASP83, PHE88, ARG89, PHE90, SER330, LEU341	−7.4

a Binding affinity [kcal/mol].

4YPF: Crystal structure of *T. cruzi* Histidyl-tRNA synthetase in complex with quinolin-3-amine.

1S0J: *Trypanosoma cruzi trans*-sialidase in complex with MuNANA (Michaelis complex).

4K32: Crystal structure of geneticin bound to the leishmanial rRNA A-site.

6QDA: Leishmania major *N*-myristoyltransferase in complex with quinazoline inhibitor IMP-0000811.

## Discussion

We have carried out an expeditious green synthesis of a series of diversely substituted benzopyrazines with a high atom economy using an ultrasonic probe. No catalyst and/or additive was used during the reaction. The reaction was conducted in the greenest solvent water and extracted with a recommended green solvent ethyl acetate (McElroy et al., 2015). The reaction comprises a nucleophilic addition of the 1,2-dicarbonyl compounds to the diamines. A plausible mechanism may involve ultrasound-assisted activation of the carbonyl groups and subsequent attack of the lone pair of electrons of the amine groups due to a negative electromeric (-E) effect. Eventually, the elimination of two water molecules and subsequent ring-closure lead to the formation of benzopyrazines with a high atom economy. Our method, as described herein, complies with most of the green chemistry principles by removing the use of hazardous solvent/catalyst/additive/procedure (Bandyopadhyay and Banik, 2021). All the reactions were carried out for 3 minutes to remove/reduce the possibility of by-product formation as the reaction mixtures became hot after sonication for an average of 3 minutes, and undesired by-products might form.

Up-/downregulation of a specific enzyme(s) (proteins) is associated with almost all diseases. Subsequently, appropriate inhibition of upregulating disease-causing enzyme(s) (direct or indirect) through enzyme-ligand interactions is considered an effective strategy for treating the specific disease. The enzyme *T. cruzi* histidyl-tRNA helps the parasite to incorporate histidine during its protein synthesis through aminoacylation reaction in a two-step process. Accordingly, inhibition of this enzyme will stop the required protein synthesis, which will kill the parasite (Pham et al., 2014). Then again, *T. cruzi trans*-sialidase is a membrane-anchored enzyme that helps the parasite to transfer sialic acids from the cellular surface of the host to that of the parasite. As no known human analog of this enzyme exists, *T. cruzi trans*-sialidase is considered a safe drug target for Chagas’ disease (Miller and Roitberg, 2013). On the other hand, the leishmanial ribosomal-RNA A-site is considered a suspected binding site for the small molecule inhibitors, which can interfere with translation processes in the course of protozoal protein synthesis (Shalev et al., 2013). In addition, another enzyme leishmania major *N*-myristoyltransferase has been reported as a validated drug target in the treatment of leishmaniasis. This enzyme acts as a biocatalyst in the co-translational N-terminal myristoylation reaction, which is essential in synthesizing a wide range of pathogenic proteins (Bell et al., 2020). Based on these published reports, the four proteins mentioned earlier were chosen as the biological targets in the present study.

Developing new therapeutic agents to counter eukaryotic pathogens is exceedingly challenging. Apart from the drug discovery-related regular hassles, this particular effort needs to balance two additional crucial factors: 1) the evolutionary conservation of drug targets between the animal or insect (host) and the microorganism and 2) the development of strain-dependent drug resistance in the human body. Chagas’ disease was mainly originated and confined to the Latin American countries until a few decades ago; however, the epidemiology of this life-threatening disease has been changing with time. An increased number of cases have been identified in the United States of America, Canada, and many other countries from Europe, some parts of Africa, the Eastern Mediterranean, and the Western Pacific regions. Currently, an estimated 75 million people are in the risk zone of this disease (Maheshwari and Bandyopadhyay, 2021; World Health Organization, 2021).

In contrast, infection due to the protozoa *Trypanosoma cruzi* is curable if appropriate treatment can be started soon after the infection. Leishmaniasis, another major vector-borne protozoal disease, has three major types: visceral, cutaneous, and mucocutaneous. An estimated 700,000 to 1 million people become infected each year (Maheshwari and Bandyopadhyay, 2021; World Health Organization, 2021). Accordingly, there is a strong need for new drugs with higher potency and far-ranging efficacy against trypanosome parasites such as Leishmania and Trypanosoma. Our benzopyrazine **1** showed comparable *in vitro* activity against both the protozoa (*L. Mexicana* and *T. cruzi*) with IC_50_ values 12.46 and 37.85 µM respectively, whereas the standard commercial drugs nifurtimox and miltefosine have IC_50_ values 9.32 and 19.56 µM against *L. Mexicana* and *T. cruzi*. Based on the observed similarities in the IC_50_ values, we hypothesized the inhibitory activity of benzopyrazine **1,** which has been validated through subsequent *in silico* protein-ligand interactions study. Two other benzopyrazines **3** and **4** showed moderate to high IC_50_ values (against the *L. mexicana* (M378) strain (50.86 and 39.83 µM, respectively) (Table 2).

**FIGURE 6:**
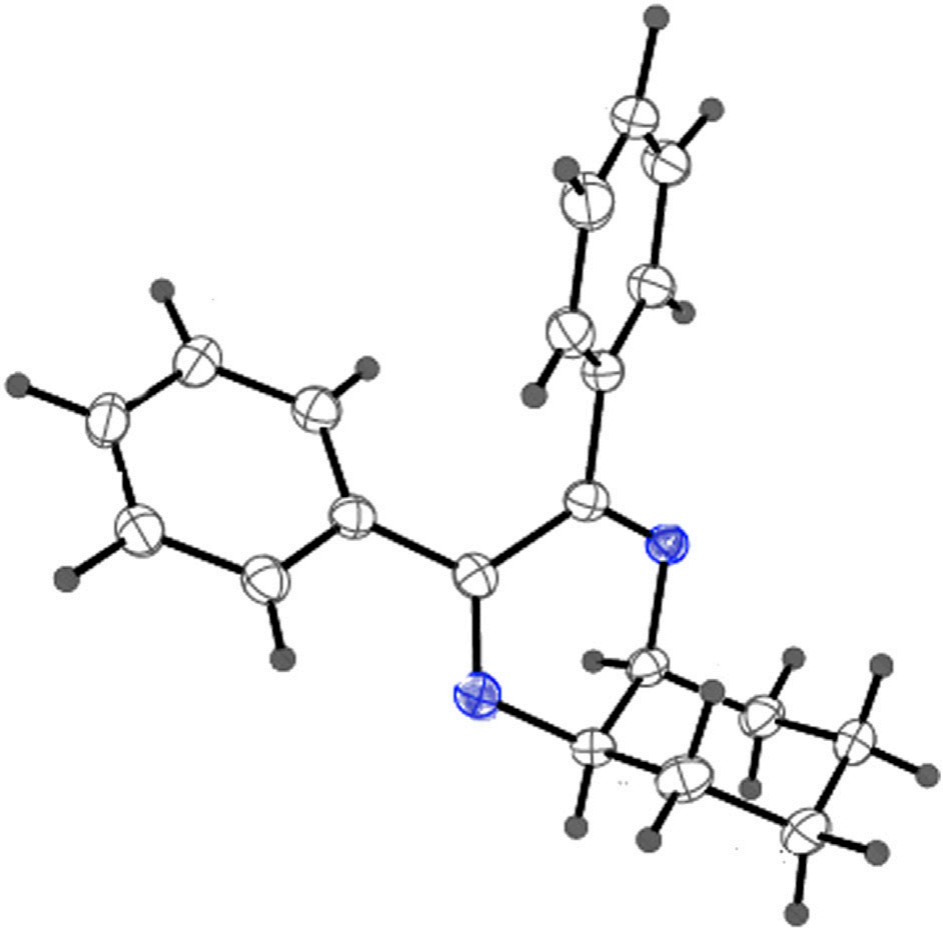
X-ray crystallographic structure (ORTEP) of Compound **2**. Thermal ellipsoids are shown with 50% probability.

As has been mentioned earlier, the proteins *T. cruzi* Histidyl-tRNA synthetase (PDB ID: 4YPF) and *T. cruzi trans*-sialidase (PDB ID: 1S0J) are considered as two major biological drug targets for Chagas’ disease, whereas Leishmanial rRNA A-site (PDB ID: 4K32) and Leishmania major *N*-myristoyltransferase (PDB ID: 6QDA) are considered as the major biomolecular drug targets for leishmaniasis (Kelly et al., 2020). Benzopyrazine **1** demonstrated better binding affinity towards all the four proteins than the standard control drug miltefosine and comparable docking scores with another control drug nifurtimox (Table 3). Benzopyrazine **1** binds the ARG156, ARG165, and HID158 residues of *T. cruzi* Histidyl-tRNA synthetase, ALA59, VAL95, LEU176, and ASP96 residues of *T. cruzi trans*-sialidase, forms salt-bridge with Leishmanial rRNA A-site, and binds the PHE 232, HID 219, GLU82, SER330 residues of the Leishmania major *N*-myristoyltransferase (Figures 2–5). The binding sites, type of interactions, hydrophobic residues, and docking scores of compound **1** with the four biomolecular targets (PDB IDs: 4YPF, 1S0J, 4K32 and 6QDA) have been summarized in Table 4. Compound 1 showed a higher binding affinity with the proteins with PDB IDs 6QDA and 1S0J (−7.4 and −7.3 kcal/mol, respectively) than the biomolecular targets with PDB IDs 4K32 and 4YPF (−6.3 and −6.7, respectively). The protein-ligand docking interactions of the co-crystallized ligands as described in the protein data bank (PDB) have also been conducted, and the results have been summarized in Supplementary Table S1 (see Supplementary material). Notably, for the protein with PDB ID 4YPF, compound **1** has a higher binding affinity (−6.7 kcal/mol) than the co-crystallized ligand quinoline-3-amine (−5.8 kcal/mol). It is worthy of mentioning that the *cis*-stereochemistry of the 5,6,7,8-tetrahydroquinoxaline ring-juncture (Compounds **1** and **2**) was confirmed by X-ray crystallographic analysis of compound **2**. The X-ray crystallographic structure (ORTEP) of Compound **2** is shown in Figure 6. The thermal ellipsoids are shown with 50% probability. The asymmetric unit comprises one molecule of compound **2** situated in a general position. The X-ray parameters can be found in Supplementary Table S2 (see Supplementary material). Here it is essential to mention that the benzopyrazines **3** and **4** demonstrated moderate leishmanicidal activity against *L. mexicana* (M378) strain. In addition, the lead compound **1** showed good leishmanicidal and trypanocidal activities (*in vitro*) against both *L. mexicana* (M378) and *T. cruzi* (NINOA) strains compared to the standard controls. The other eight analogs did not demonstrate notable biological activity against any of the tested strains. This observation indirectly supports the selective toxicity of **1**, **3**, and **4** against the pathogenic strains. If all compounds showed activity against the parasites, this effect could be considered a toxic effect.

Finally, the drug-likeness of all the eleven benzopyrazine derivatives has been determined following the RO5 (Lipiniski et al., 2001). Seven out of eleven compounds did not show any violations which support the drug-likeness of these molecules. Benzopyrazines **1**, **3**, and **4** are in good agreement with the RO5 (Table 5). Based on the promising druglike-ness as shown in Table 5, it is expected that the molecules should have good pharmacokinetics and pharmacodynamics properties.

**TABLE 5:** Validation^†^ of drug-likeness of the benzopyrazines (**1**–**11**).

Compound	miLogP^a^	HBA^b^	HBD^c^	TPSA^d^	RB^e^	MW^f^	Violation
**1**	2.96	2	0	24.73	1	212.30	0
**2**	4.62	2	0	24.73	2	288.39	0
**3**	3.41	2	0	25.78	1	206.25	0
**4**	3.44	3	0	35.02	2	236.27	0
**5**	4.06	2	0	25.78	1	240.69	0
**6**	3.34	5	0	71.61	2	251.25	0
**7**	5.91	5	0	71.61	3	355.40	1
**8**	5.34	5	0	71.61	3	363.32	1
**9**	3.56	5	0	71.61	2	265.27	0
**10**	5.08	2	0	25.78	2	282.35	1
**11**	5.01	5	0	71.61	3	327.34	1

^†^ Molinspiration property engine v2018.10.

a miLogP: Moriguchi octanol-water partition coefficient, is based on quantitative structure-LogP relationships, by using topological indexes.

b Hydrogen bond acceptor.

c Hydrogen bond donor.

d Total polar surface area.

e Number of rotatable bonds.

## Conclusion

An expeditious green ultrasound-assisted one-pot procedure of synthesizing benzopyrazines is reported. This newly developed method satisfies many aspects of green chemistry and maintains a high atom economy. No catalyst/support/additive/hazardous solvents were used to accomplish the synthesis. Most of the synthesized compounds possess drug-likeness and follow RO5. A minor violation of RO5 was noticed for four out of eleven products. Benzopyrazines **3** and **4** demonstrated moderate leishmanicidal activity against *L. mexicana* (M378) strain. The selective lead compound **1** showed good leishmanicidal, and trypanocidal activities (*in vitro*) against both *L. mexicana* (M378) and *T. cruzi* (NINOA) strains compared to the standard controls. The hypothesis of binding of the lead benzopyrazine **1** to the active sites of the four proteins (*T. cruzi* Histidyl-tRNA synthetase, *T. cruzi trans*-sialidase, leishmanial rRNA A-site, and leishmania major *N*-myristoyltransferase) responsible for Chagas’ disease and leishmaniasis disease. As mentioned earlier, the inhibition of the proteins with the compounds **1** has been validated by *in silico* molecular docking studies. Accordingly, benzopyrazine **1,** as reported herein, may find its application in the future drug development process against two major neglected tropical diseases: Chagas’ disease and leishmaniasis.

## Data Availability

The original contributions presented in the study are included in the article/Supplementary Material, further inquiries can be directed to the corresponding authors.
