# Supplementary material for: Benzopyrazine-Based Small Molecule Inhibitors As Trypanocidal and Leishmanicidal Agents: Green Synthesis, In Vitro, and In Silico Evaluations
Source: Front Chem. 2021 Sep 17;9:725892. doi: 10.3389/fchem.2021.725892 (PMC8484882; doi:10.3389/fchem.2021.725892)
Supplement: Supplementary file 1 [file DataSheet1.PDF]

Supplementary Material

Benzopyrazine-Based Small Molecule Inhibitors As Trypanocidal And Leishmanicidal Agents: Green synthesis, *in vitro*, and *in silico* Evaluations

Jonathan Rock, Daniel Garcia, Omar Espino, Shaila A. Shetu, Manuel J. Chan-Bacab, Rosa Moo-Puc, Navin B. Patel, Gildardo Rivera*, Debasish Bandyopadhyay*

The ^{1}H- and ^{13}C-NMR spectra of the compounds, the docking of the four biomolecular targets with respective co-crystallized ligand and X-ray crystallographic information of Compound **2** can be found in the sequel.

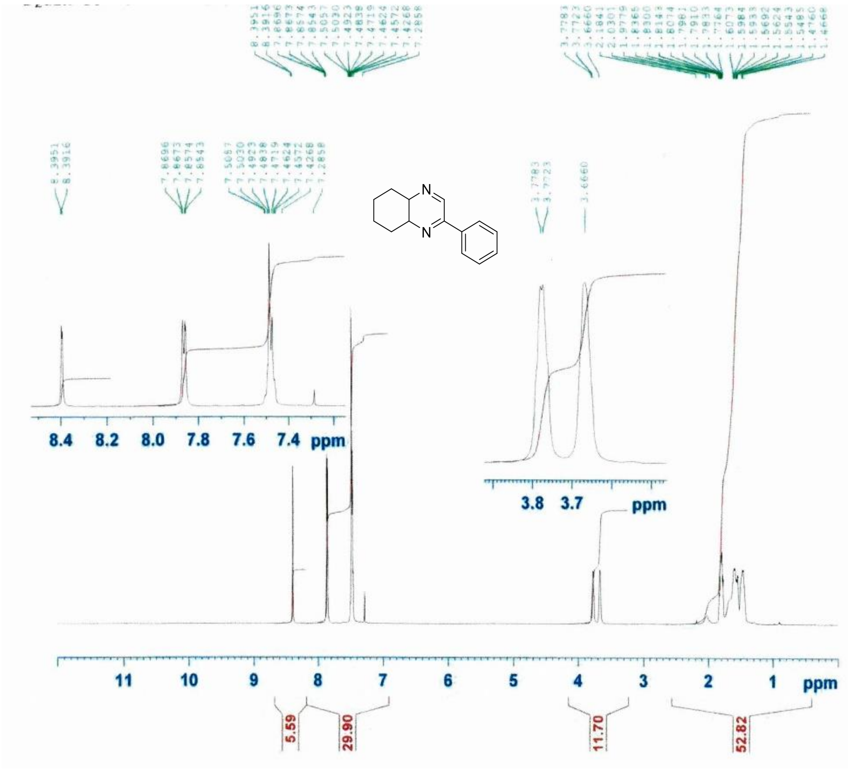

Supplementary Figure 1. ^{1}H-NMR spectrum of Compound **1** in $CDCl_3$

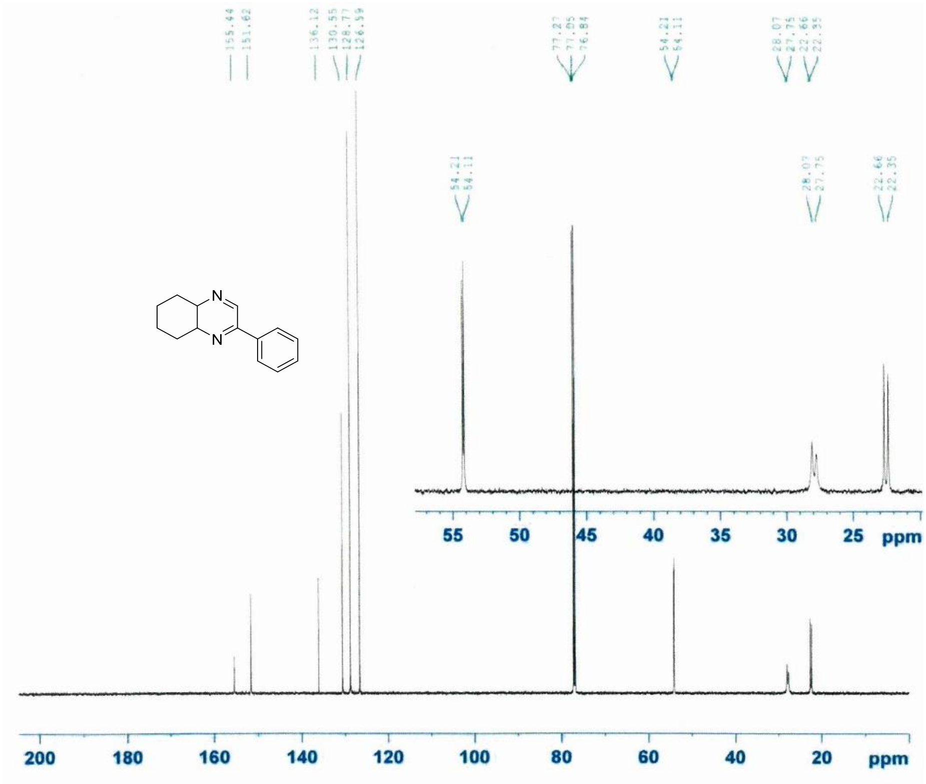

Supplementary Figure 2. ^{13}C-NMR spectrum of Compound **1** in $CDCl_3$

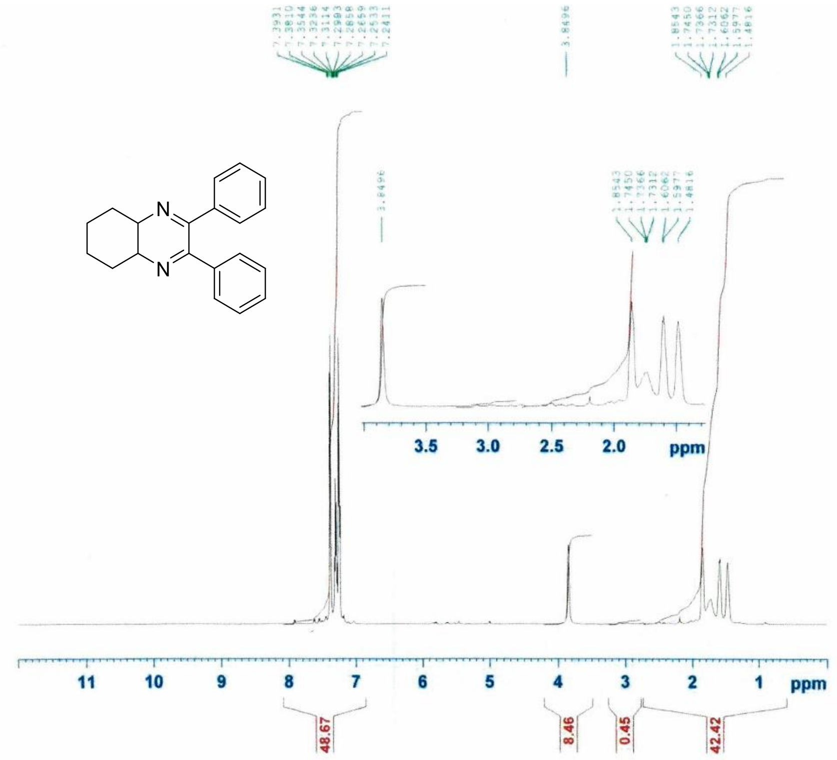

Supplementary Figure 3. ^{1}H-NMR spectrum of Compound **2** in $CDCl_3$

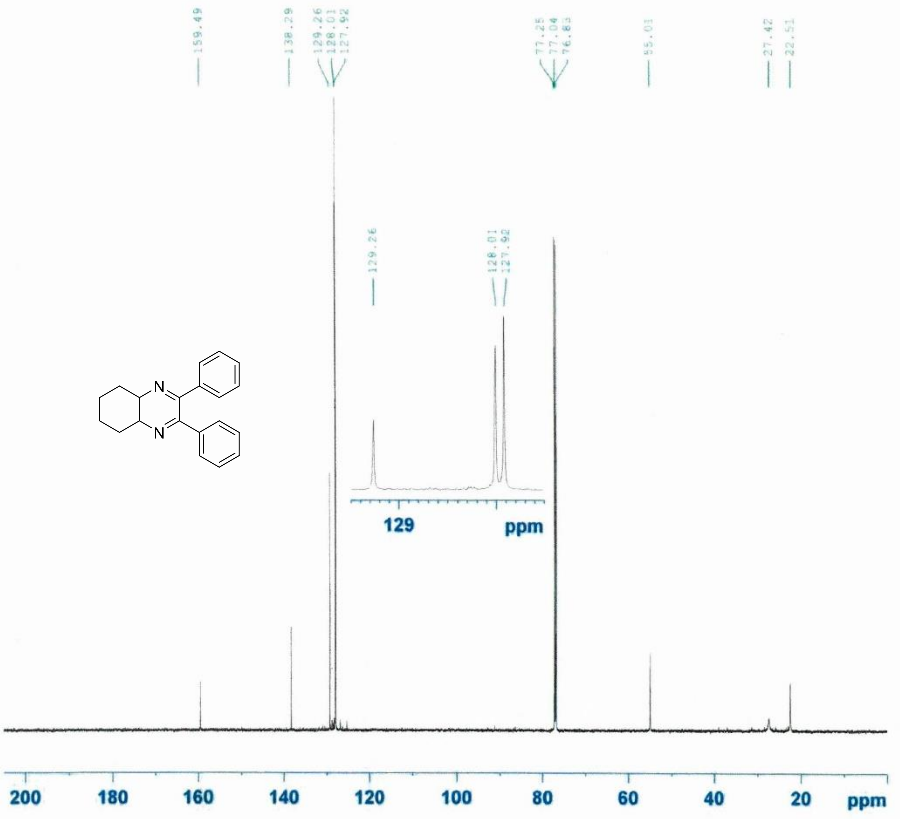

Supplementary Figure 4. ^{13}C-NMR spectrum of Compound **2** in $CDCl_3$

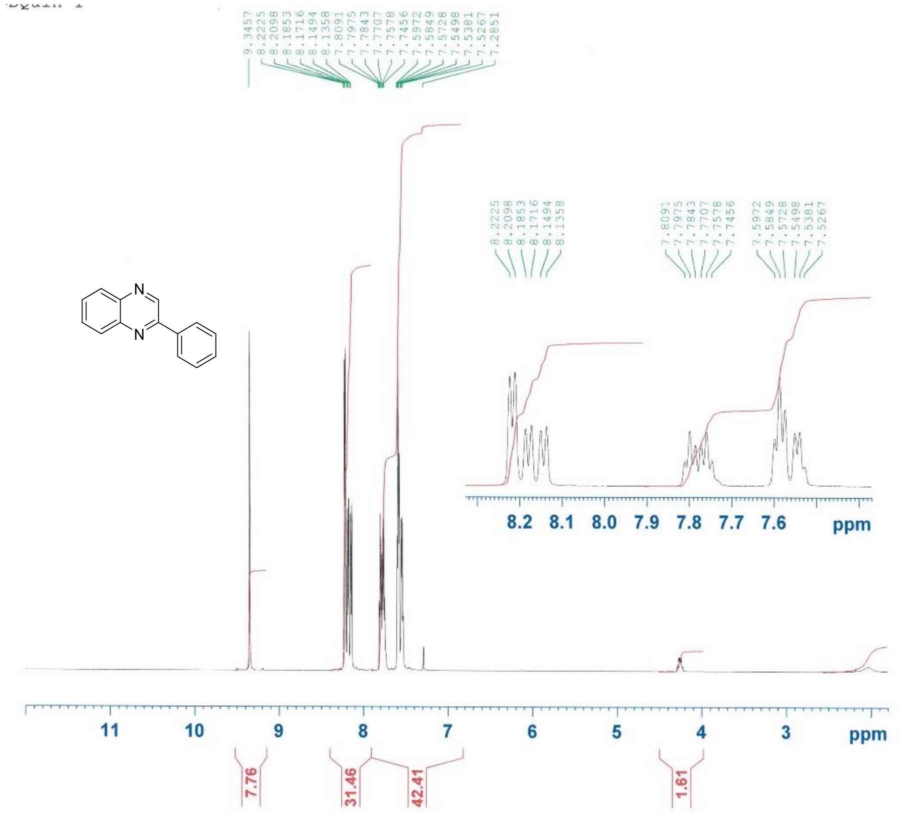

Supplementary Figure 5. ^{1}H-NMR spectrum of Compound **3** in $CDCl_3$

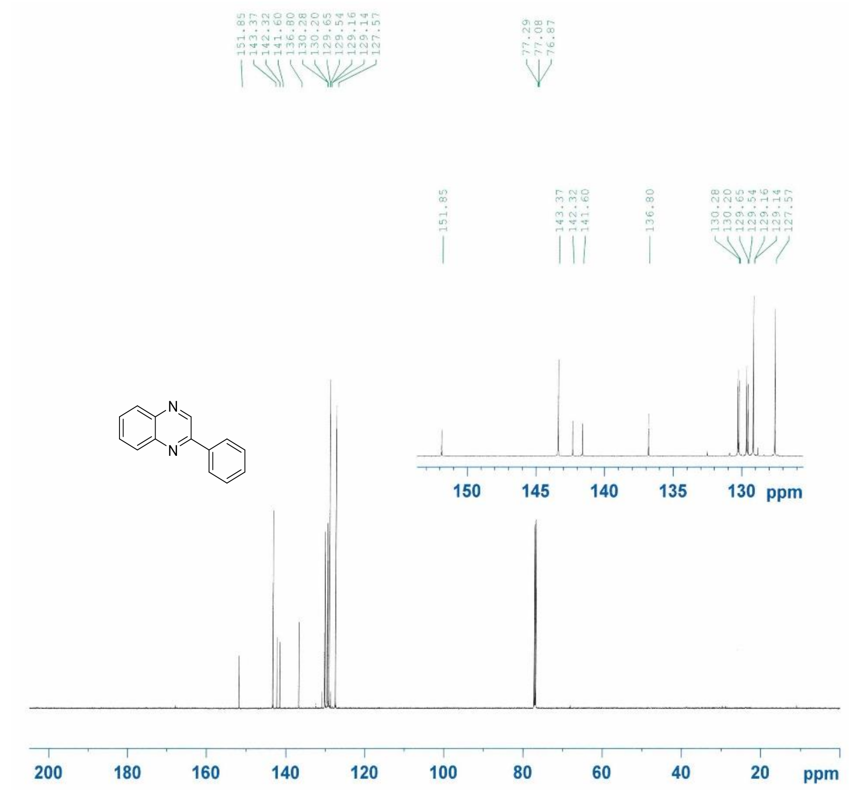

Supplementary Figure 6. ^{13}C-NMR spectrum of Compound **3** in $CDCl_3$

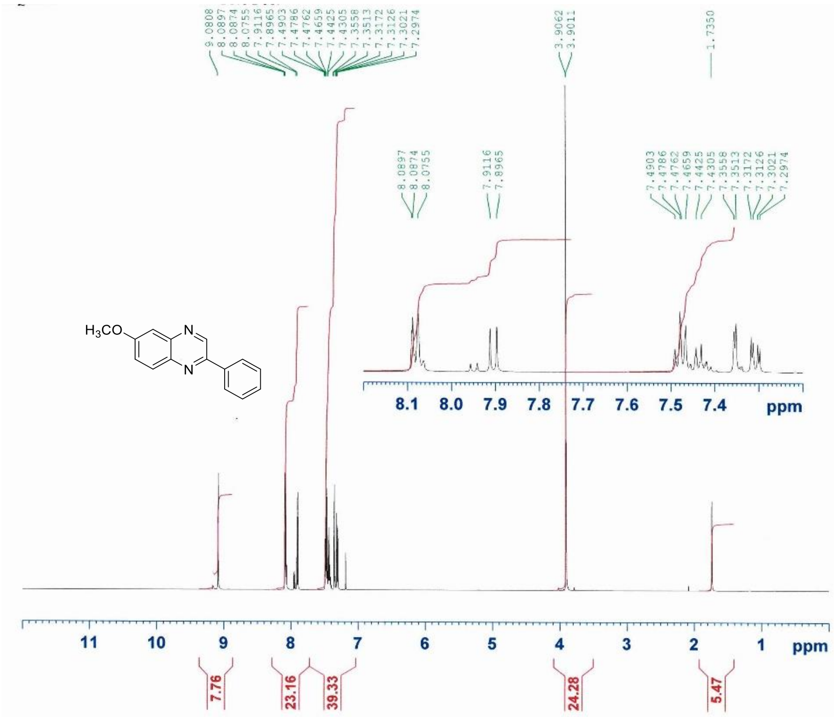

Supplementary Figure 7. ^{1}H-NMR spectrum of Compound **4** in $CDCl_3$

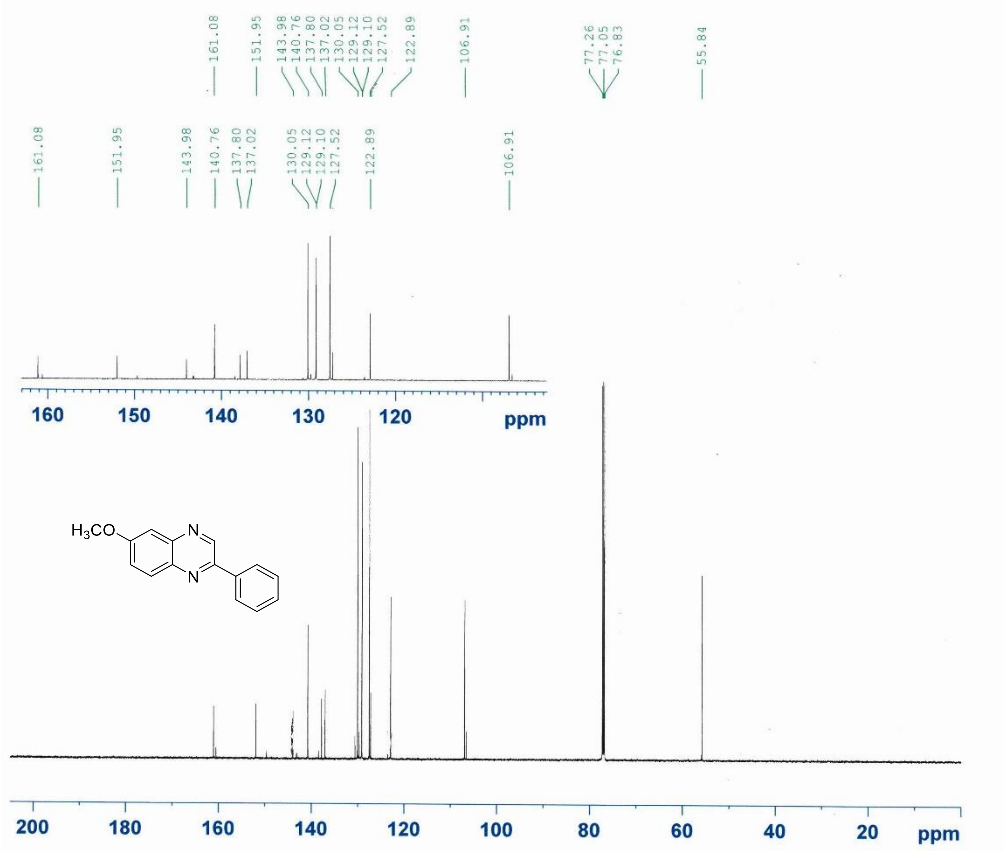

Supplementary Figure 8. ^{13}C-NMR spectrum of Compound **4** in $CDCl_3$

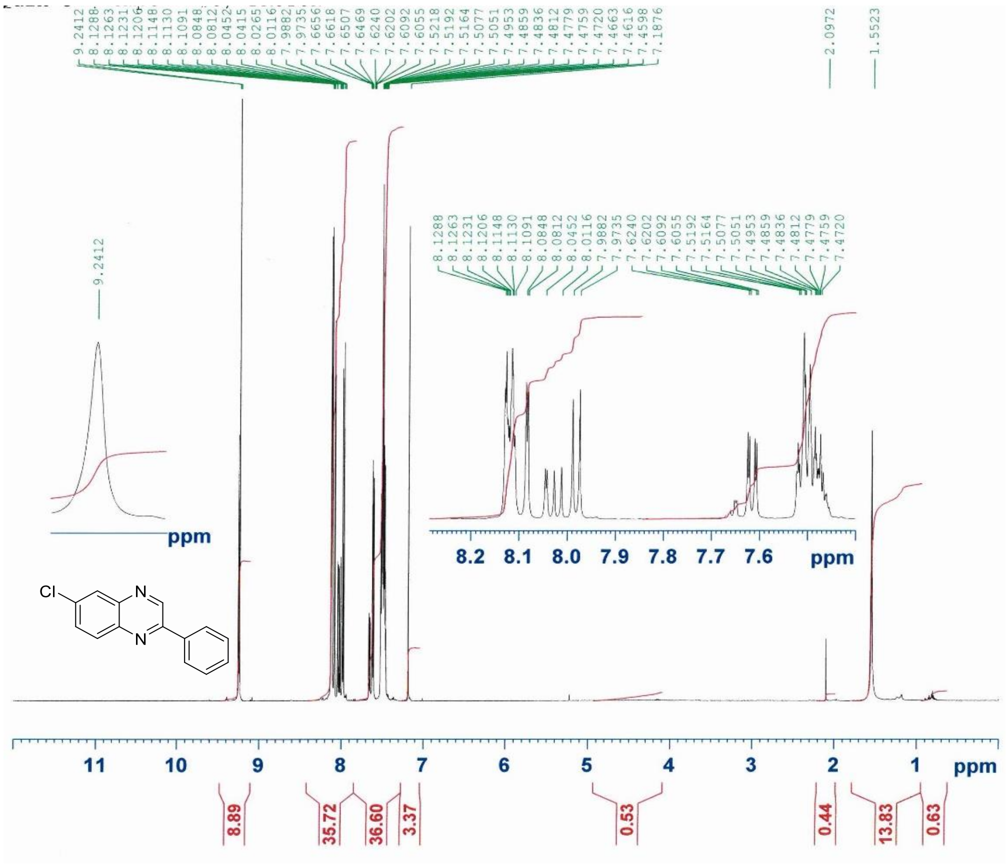

Supplementary Figure 9. ^{1}H-NMR spectrum of Compound **5** in $CDCl_3$

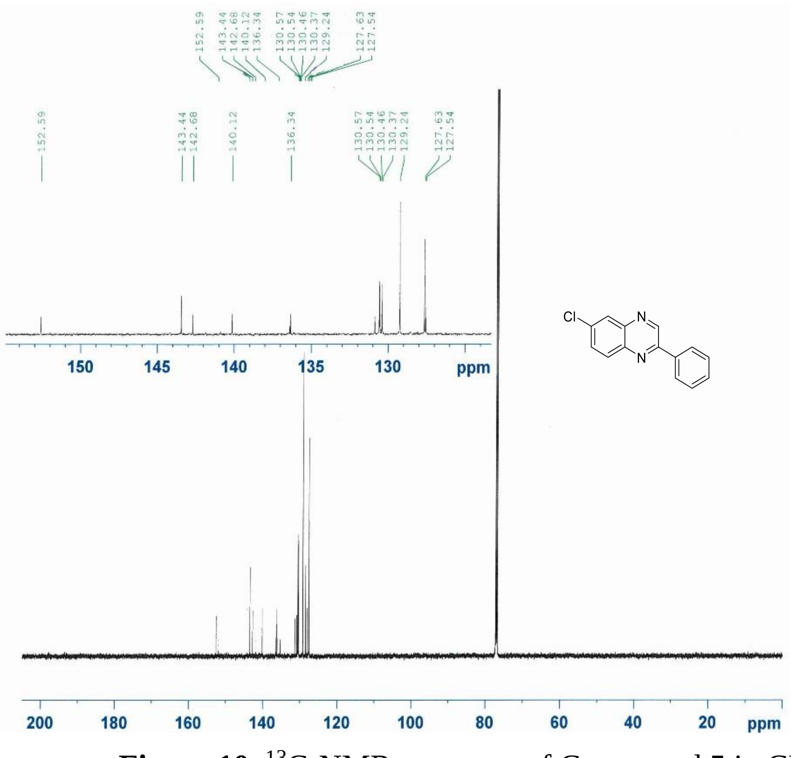

Supplementary Figure 10. ^{13}C-NMR spectrum of Compound **5** in $CDCl_3$

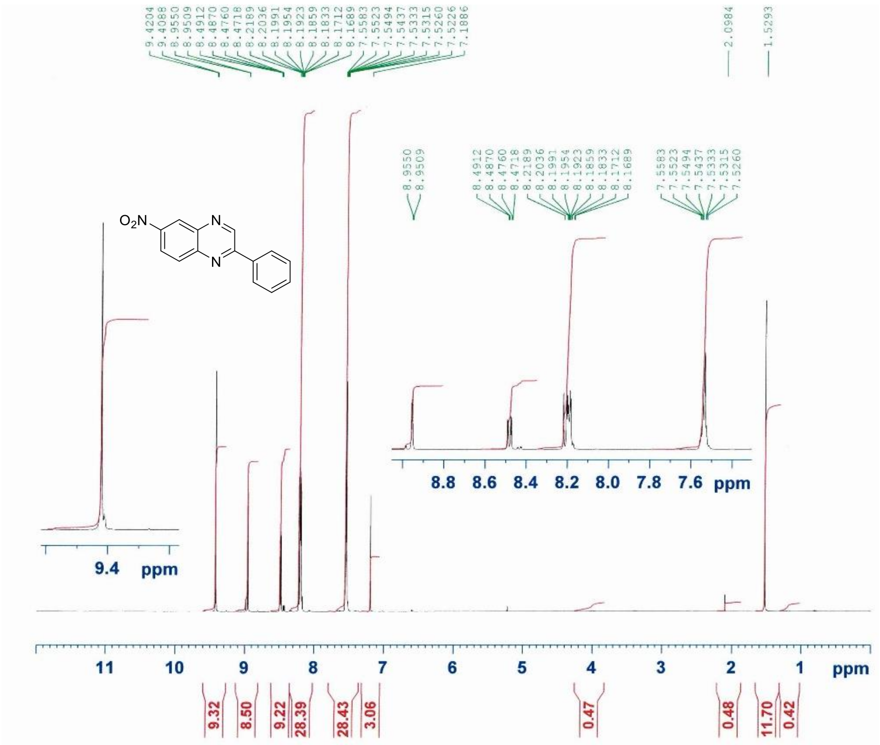

Supplementary Figure 11. ^{1}H-NMR spectrum of Compound **6** in $CDCl_3$

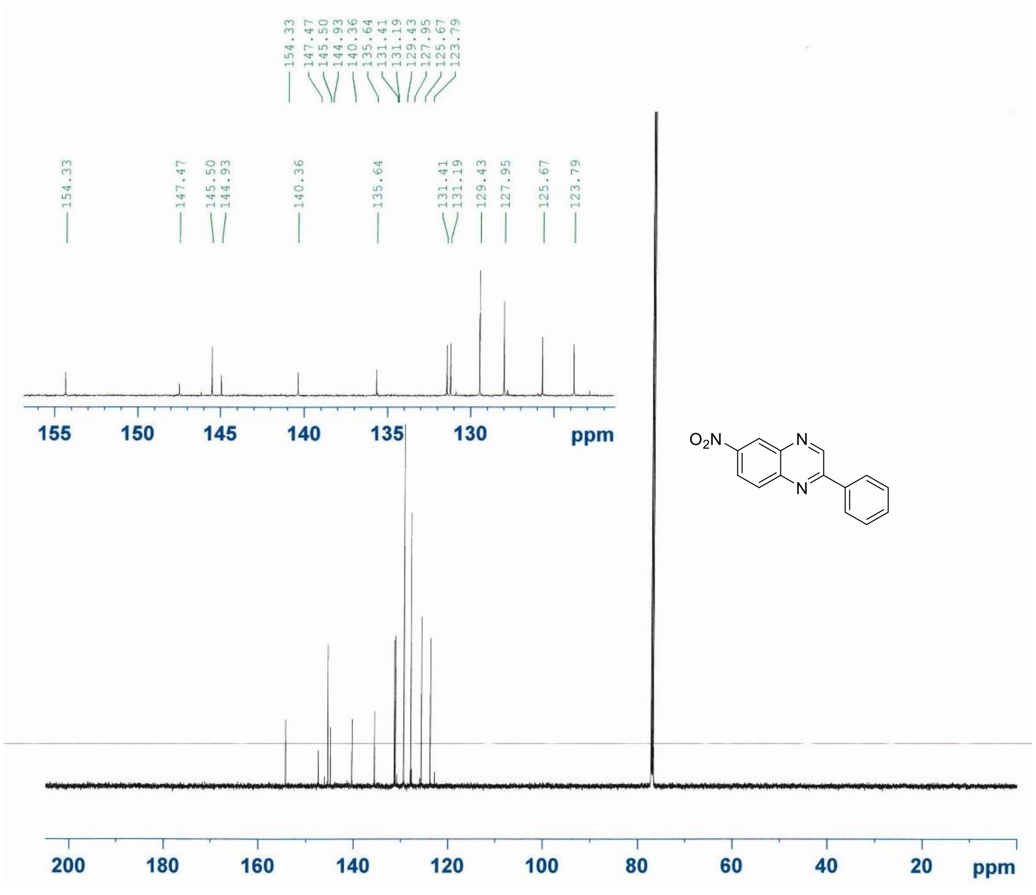

Supplementary Figure 12. ^{13}C-NMR spectrum of Compound **6** in $CDCl_3$

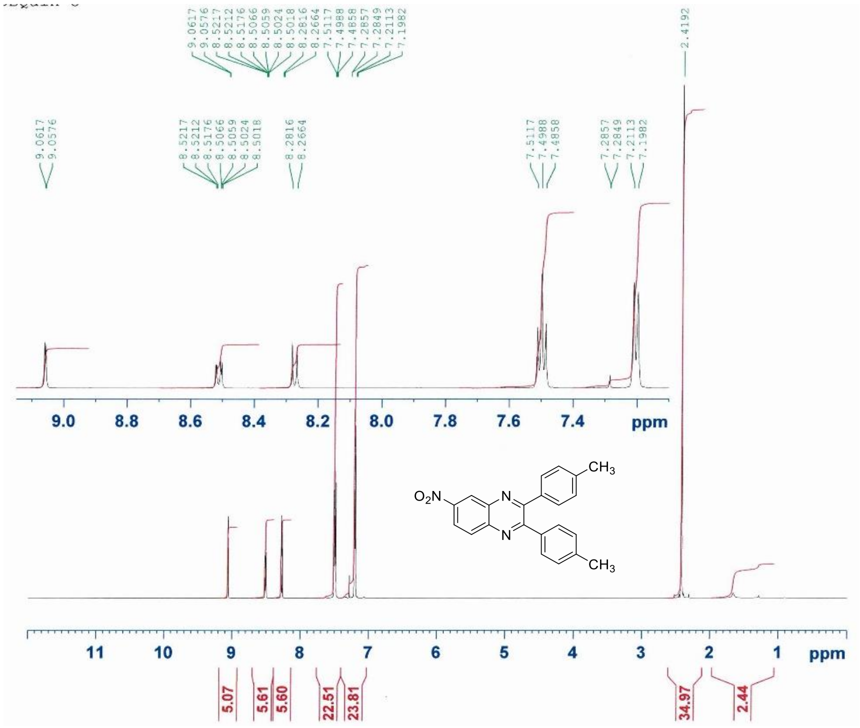

Supplementary Figure 13. ^{1}H-NMR spectrum of Compound **7** in $CDCl_3$

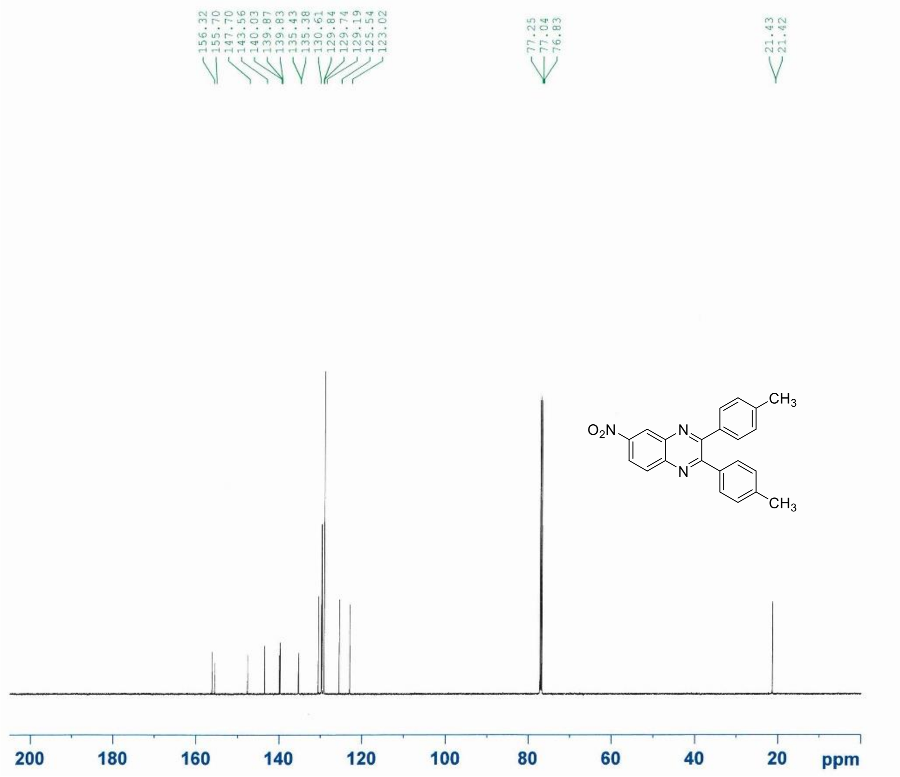

Supplementary Figure 14. ^{13}C-NMR spectrum of Compound **7** in $CDCl_3$

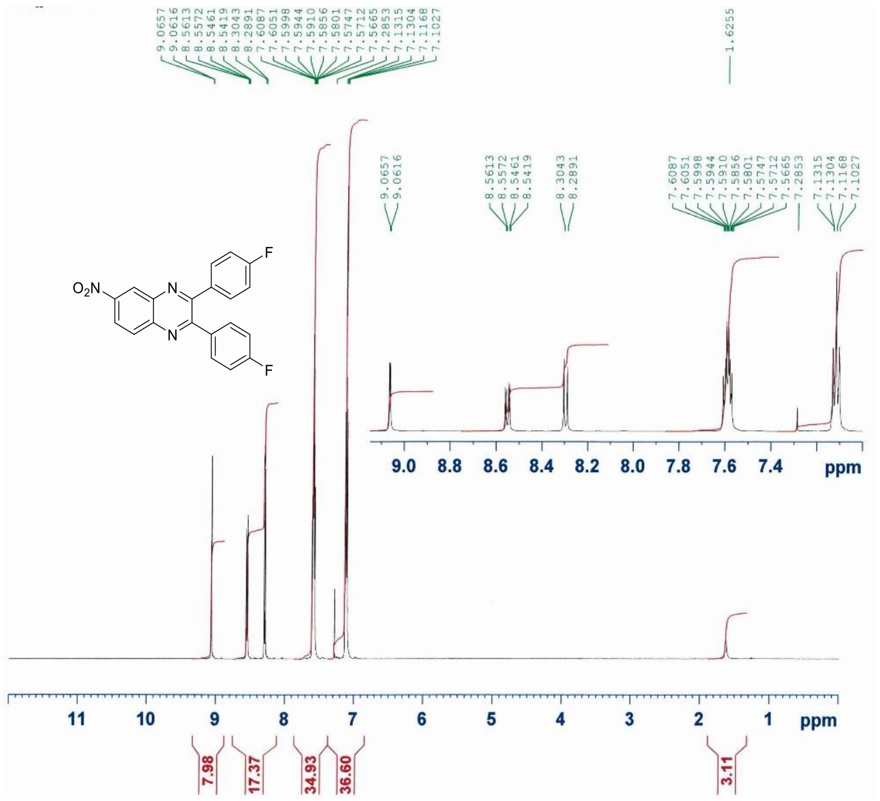

Supplementary Figure 15. ^{1}H-NMR spectrum of Compound **8** in $CDCl_3$

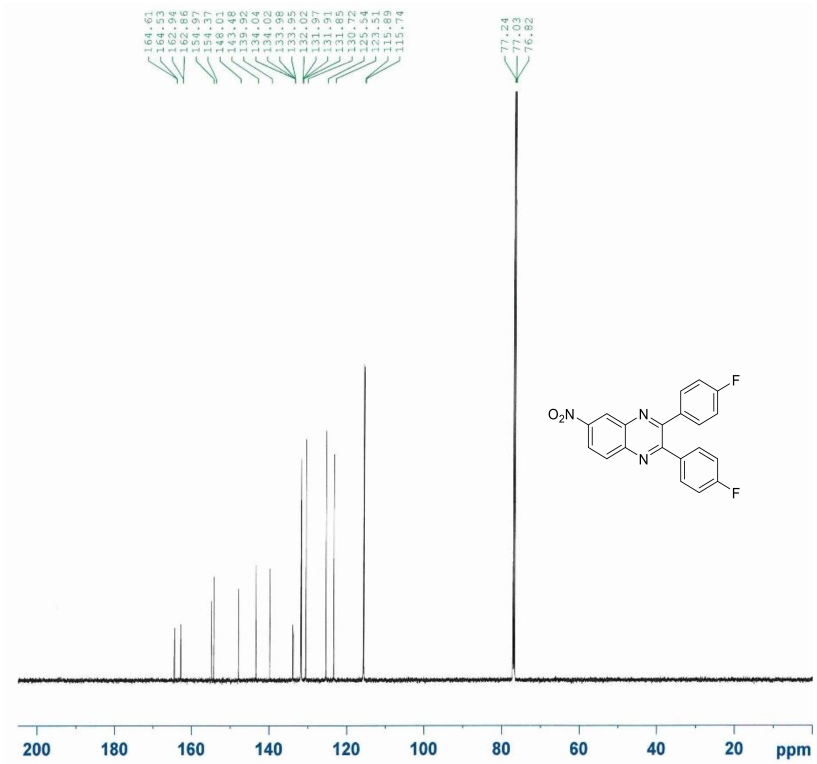

Supplementary Figure 16. ^{13}C-NMR spectrum of Compound **8** in $CDCl_3$

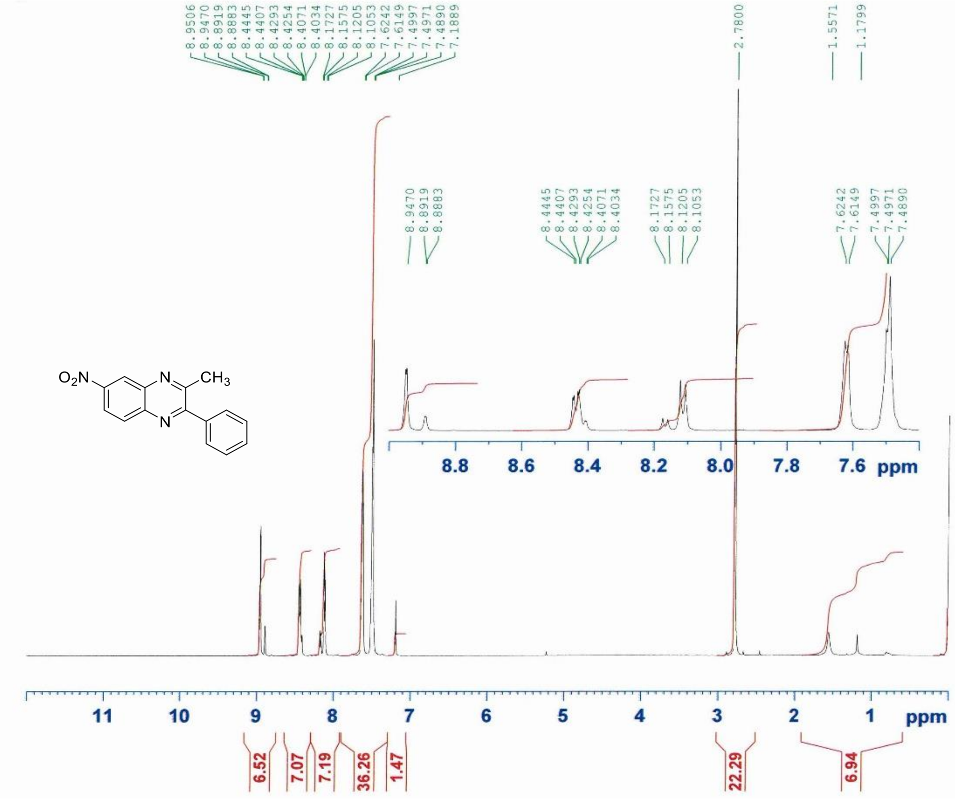

Supplementary Figure 17. ^{1}H-NMR spectrum of Compound **9** in $CDCl_3$

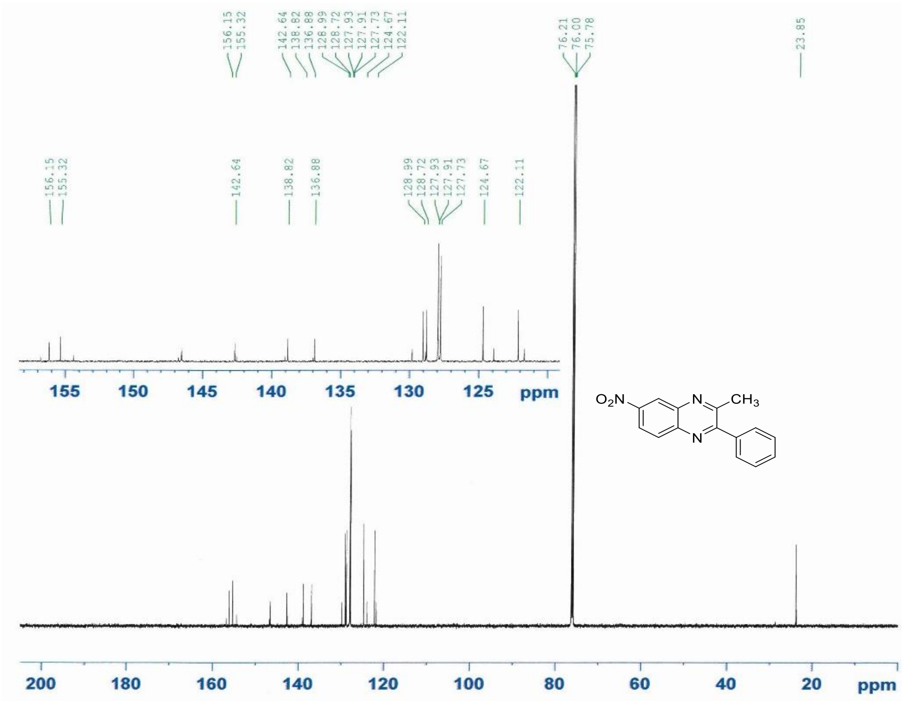

Supplementary Figure 18. ^{13}C-NMR spectrum of Compound **9** in $CDCl_3$

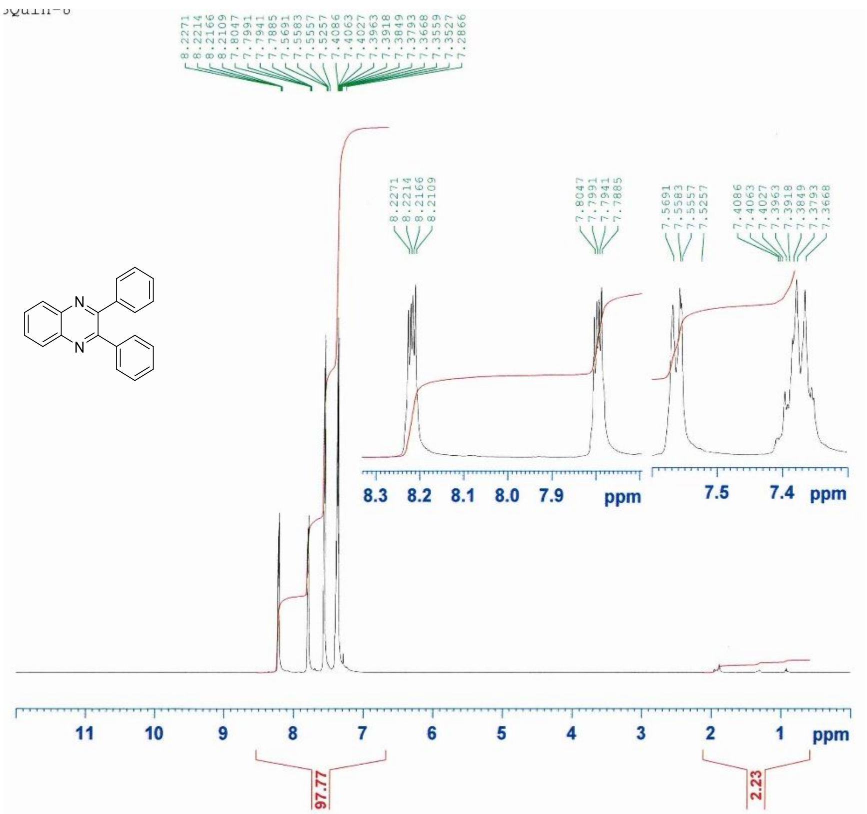

Supplementary Figure 19. ^{1}H-NMR spectrum of Compound **10** in $CDCl_3$

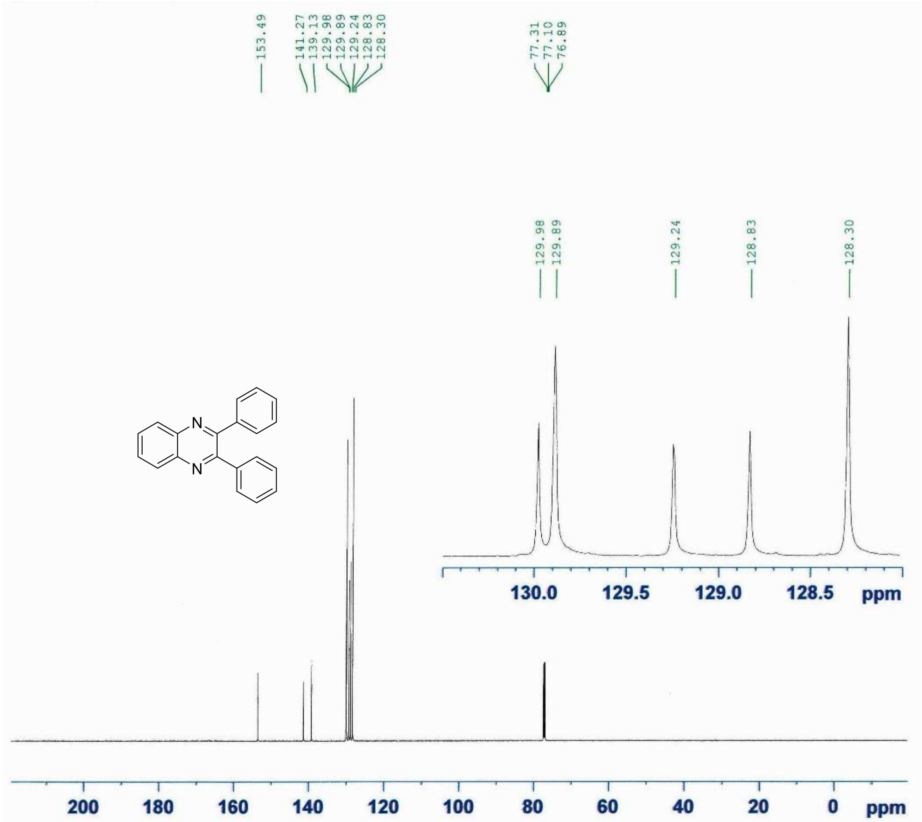

Supplementary Figure 20. ^{13}C-NMR spectrum of Compound **10** in $CDCl_3$

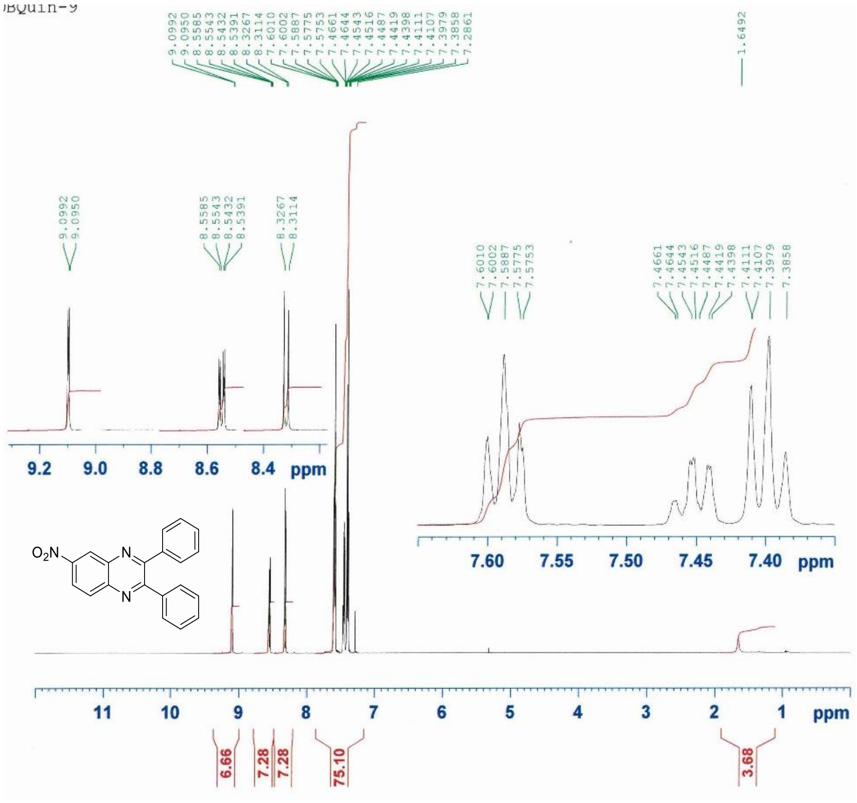

Supplementary Figure 21. ^{1}H-NMR spectrum of Compound **11** in $CDCl_3$

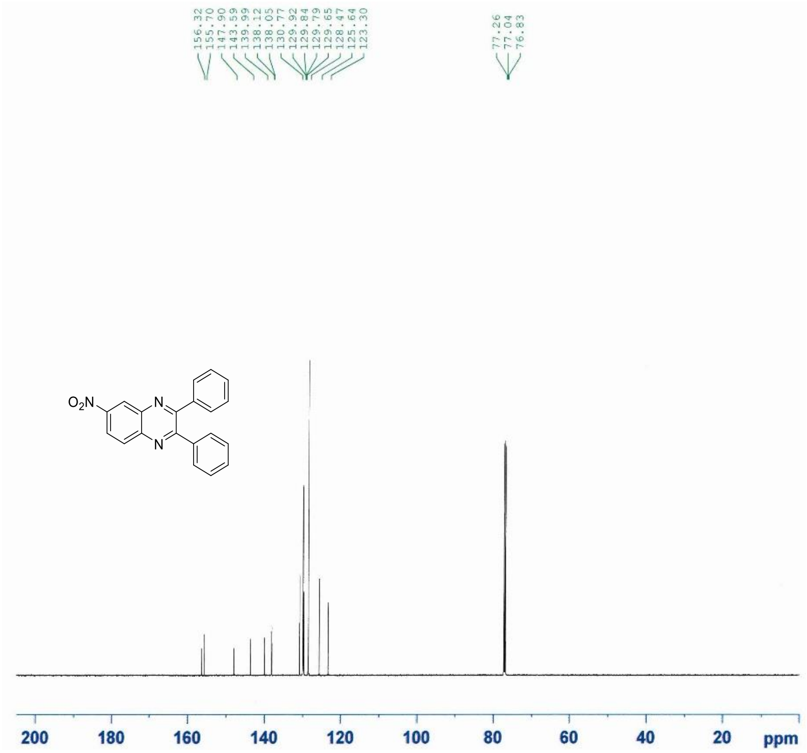

Supplementary Figure 22. ^{13}C-NMR spectrum of Compound **11** in $CDCl_3$

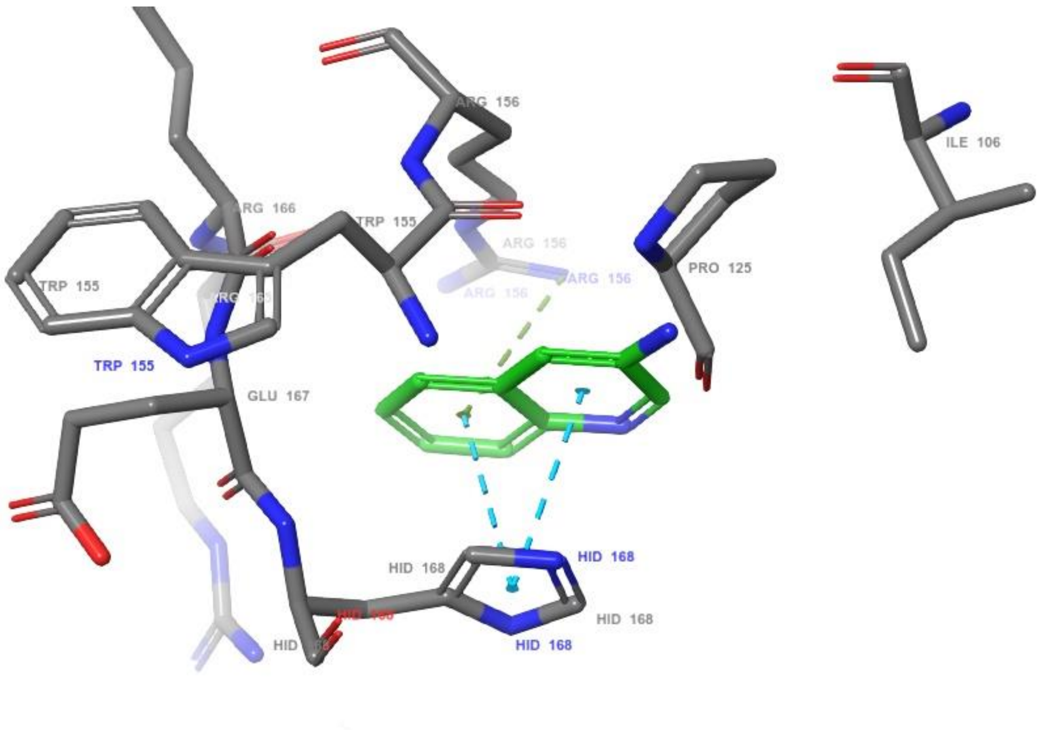

Supplementary Figure 23. Binding mode of the interactions between the co-crystallized ligand with *T. cruzi* Histidyl-tRNA synthetase (PDB ID: 4YPF)

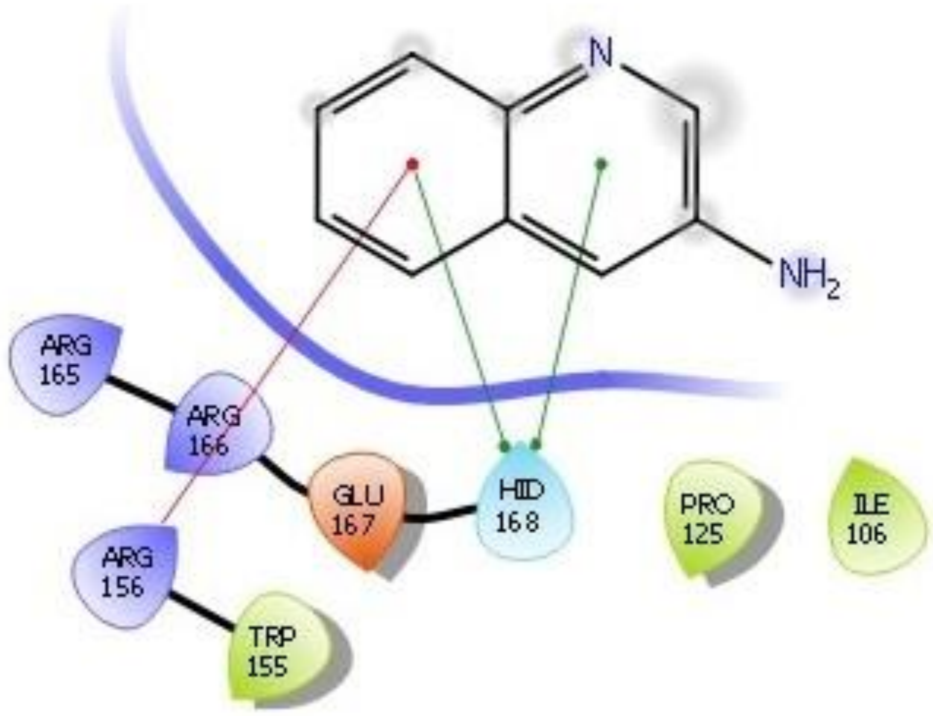

Supplementary Figure 24. Results of the validation of the co-crystallized ligand with *T. cruzi* Histidyl-tRNA synthetase active sites

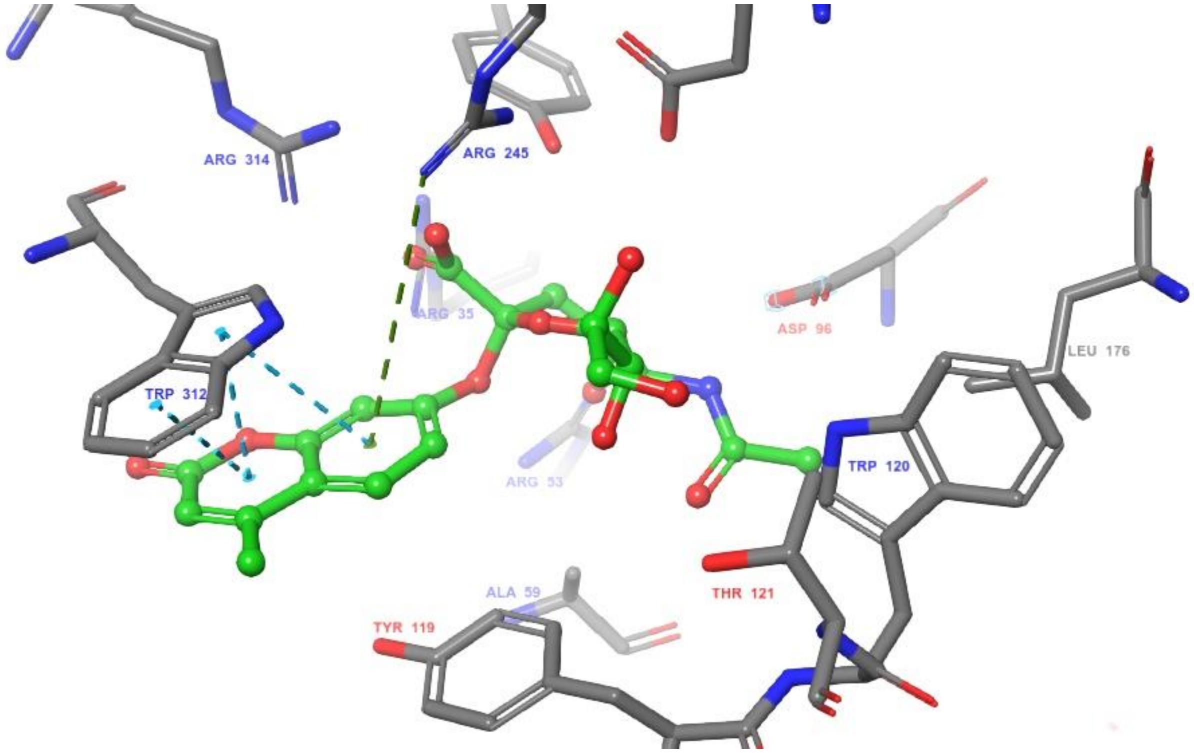

Supplementary Figure 25. Binding mode of the interactions between co-crystallized ligand with *T. cruzi trans*-sialidase (PDB ID: 1S0J)

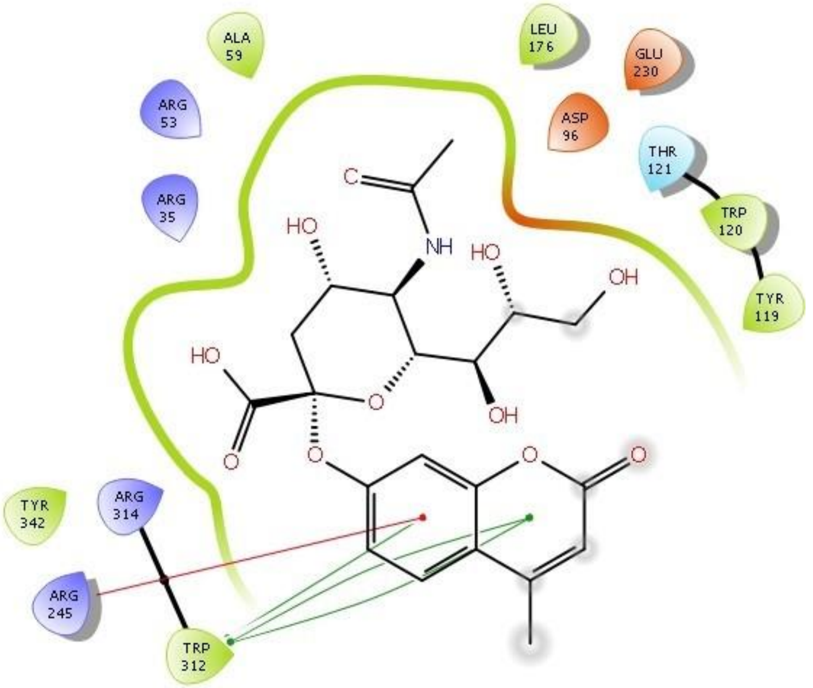

Supplementary Figure 26. Results of the validation of the co-crystallized ligand with *T. cruzi trans*-sialidase active sites

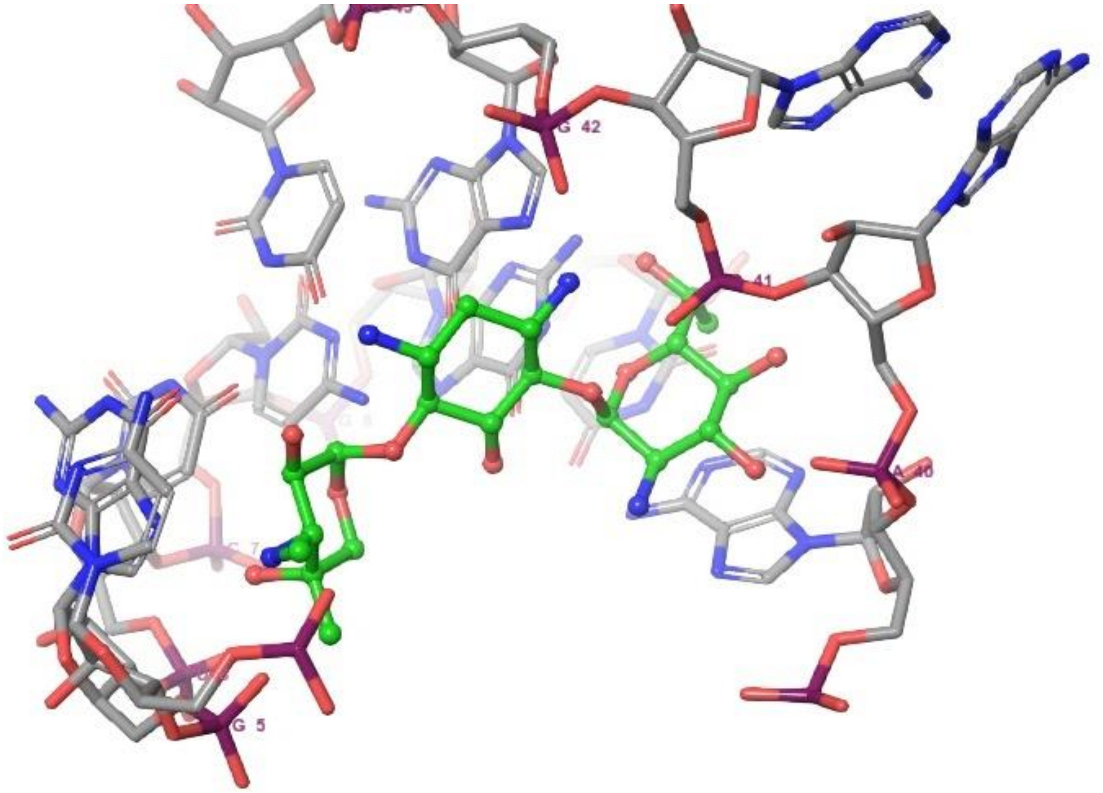

Supplementary Figure 27. Binding mode of the interactions between the co-crystallized ligand with Leishmanial rRNA A-site (PDB ID: 4K32)

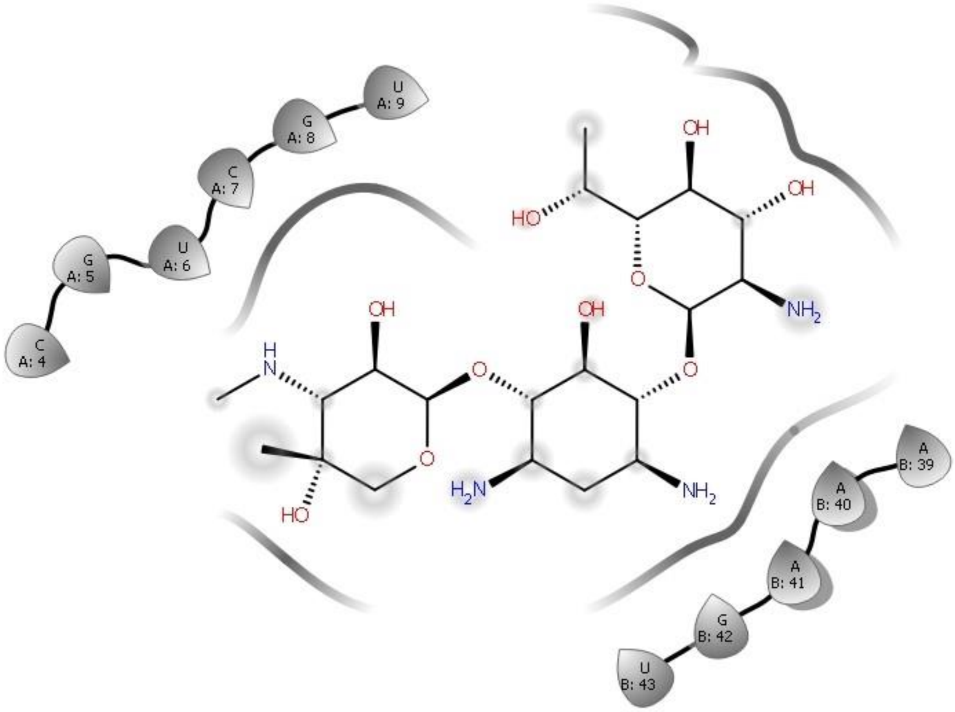

Supplementary Figure 28. Results of the validation of co-crystallized ligand with Leishmanial rRNA A-site active sites

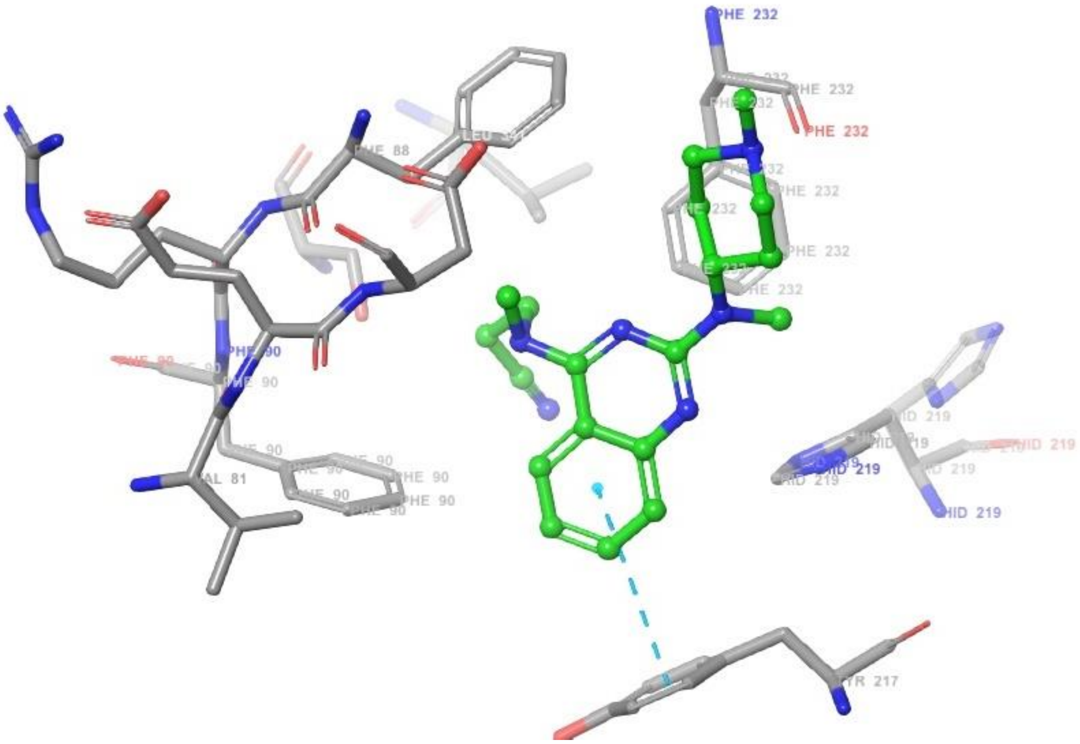

Supplementary Figure 29. Binding mode of the interactions between the co-crystallized ligand with Leishmania major *N*-myristoyltransferase (PDB ID: 6QDA)

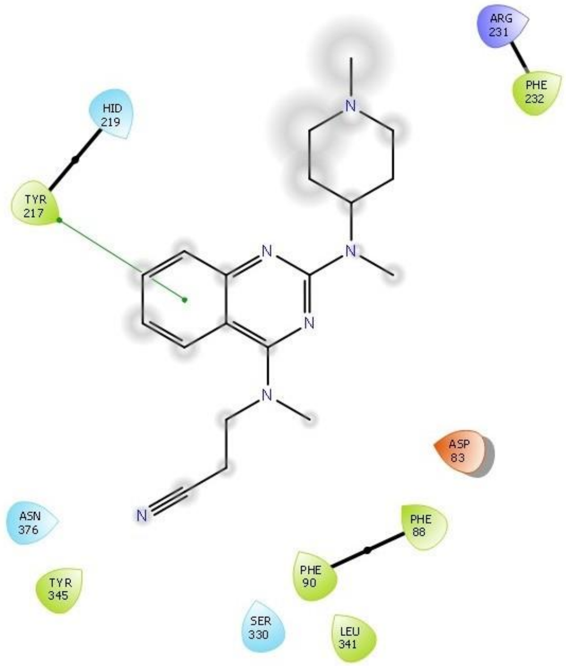

Supplementary Figure 30. Results of the validation of co-crystallized ligand with Leishmania major *N*-myristoyltransferase active sites

Supplementary Table 1. Molecular docking scores of the co-crystallized ligands with four biomolecular targets (PDB IDs: 4YPF, 1S0J, 4K32& 6QDA).

PDB ID	Structure of the co-crystallization ligand	Hydrophobic Residues	Docking score [Binding affinity [kcal/mol]	Type of Interactions	Binding Site Residue(s)
4YPF	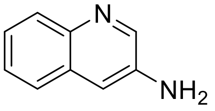 quinolin-3-amine	GLU105, ILE106, GLN109, PRO125, TRP155, ARG165, GLU167	-5.8	π- cation Stacking<br>π- π Stacking	ARG156<br>HID168
1S0J	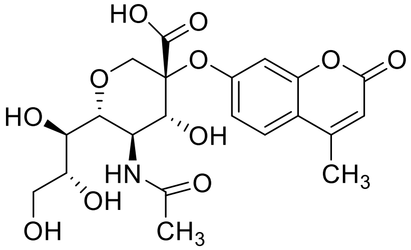	ARG35, ARG53, ALA59, ASN60, ARG93, ASP96, TYR119, TRP120, THR121, LEU176, GLU230, ARG314, TYR342	-8.1	π- π Stacking<br>π- cation Stacking	TRP 312<br>ARG245
4K32	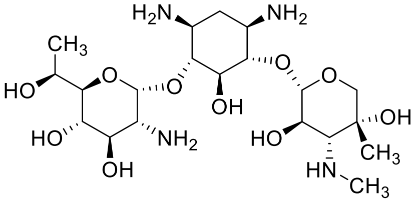	C4, G5, U6, C7, G8, U9, A39, A40, A41, G42, U43	-7.8	N/A	N/A
6QDA	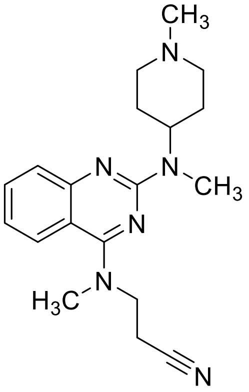	VAL81, GLU82, ASP83, PHE88, PHE90, HID219, ARG231, PHE232, SER330, LEU341, TYR345, AS376	-8.0	H-Bond<br>π- π Stacking	TYR 217

Supplementary Table 2. X-ray **c**rystallographic information of the Compound **2***

CCDC	905113
Empirical formula	$C_{20}H_{20}N_2$
Formula weight	288.38
Crystal system	orthorhombic
Space group	$P2_12_12_1$
a(Å)	6.291(3)
b(Å)	13.381(6)
c(Å)	19.082(8)
α(°)	90
β(°)	90
γ(°)	90
Volume(Å^3)	1606.3(12)
Z, Z'	4,1
ρ(calc.)	1.192
λ	0.71073
Temp.(K)	98(2)
F(000)	616
μ(mm^{-1})	0.070
T_{min}, T_{max}	0.7539, 1.000
$2\theta_{range}$(°)	2.13 to 27.49
Reflections Collected	11519
Independent reflections	2131 [R(int) = 0.0487]
Data / restraints / parameters	2131 / 0 / 259
$wR(F^2$ all data)	0.1134
$R(F$ obsd data)	0.0491
GOOF on F^2	1.009
Observed data [I > 2σ(I)]	2030
Largest and mean shift / s.u.	0.000/ 0.000

*The crystallographic data of compound 2 have been deposited with Cambridge Crystallographic Data Centre as CCDC 905113. Copies of the data can be obtained, free of charge, on application to CCDC, 12 Union Road, Cambridge CB2 IEZ, UK (fax: +44 (0)1223 336033 or e-mail: deposit@ccdc.cam.ac.uk).
